# The circular RNA circ-ERBIN promotes growth and metastasis of colorectal cancer by miR-125a-5p and miR-138-5p/4EBP-1 mediated cap-independent HIF-1α translation

**DOI:** 10.1186/s12943-020-01272-9

**Published:** 2020-11-23

**Authors:** Liang-Yan Chen, Lian Wang, Yue-Xiang Ren, Zheng Pang, Yao Liu, Xiao-Dong Sun, Jian Tu, Zheng Zhi, Yan Qin, Li-Na Sun, Jian-Ming Li

**Affiliations:** 1 Department of Pathology and Pathophysiology, Soochow University Medical School, Suzhou, 215123 People’s Republic of China; 2 Department of Pathology, the Second Affiliated Hospital of Soochow University, Suzhou, 215004 People’s Republic of China; 3 Department of Pathology, the Affiliated Hospital of Jiangnan University, Wuxi 4th People’s Hospital, Wuxi, 214062 People’s Republic of China; 4 Department of Pathology, Sun Yat-sen Memorial Hospital, Sun Yat-sen University, Guangzhou, 510120 China

**Keywords:** Colorectal cancer, Circular RNA, hypoxia, miRNA sponge

## Abstract

**Background:**

Circular RNA (circRNAs) and hypoxia have been found to play the key roles in the pathogenesis and progression of cancer including colorectal cancer (CRC). However, the expressions and functions of the specific circRNAs in regulating hypoxia-involved CRC metastasis, and the circRNAs that are relevant to regulate HIF-1α levels in CRC remain elusive.

**Methods:**

qRT-PCR was used to detect the expression of circRNAs and mRNA in CRC cells and tissues. Fluorescence in situ hybridization (FISH) was used to analyze the location of circ-ERBIN. Function-based experiments were performed using circ-ERBIN overexpression and knockdown cell lines in vitro and in vivo, including CCK8, colony formation, EdU assay, transwell, tumor growth and metastasis models. Mechanistically, luciferase reporter assay, western blots and immunohistochemical stainings were performed.

**Results:**

Circ-Erbin was highly expressed in the CRC cells and Circ-Erbin overexpression facilitated the proliferation, migration and metastasis of CRC in vitro and in vivo. Notably, circ-Erbin overexpression significantly promoted angiogenesis by increasing the expression of hypoxia induced factor (HIF-1α) in CRC. Mechanistically, circ-Erbin accelerated a cap-independent protein translation of HIF-1α in CRC cells as the sponges of miR-125a-5p and miR-138-5p, which synergistically targeted eukaryotic translation initiation factor 4E binding protein 1(4EBP-1).

**Conclusions:**

Our findings uncover a key mechanism for circ-Erbin mediated HIF-1α activation by miR-125a-5p-5p/miR-138-5p/4EBP-1 axis and circ-ERBIN is a potential target for CRC treatment.

**Supplementary Information:**

**Supplementary information** accompanies this paper at 10.1186/s12943-020-01272-9.

## Introduction

CircRNAs are a class of abundant and ubiquitous noncoding RNA molecules that been found recently in eukaryotic cells [1, 2]. Unlike other RNAs, the absence of both 5′ caps and 3′ tails allows circRNAs resisting RNases digestion, which gives them higher stability compared with linear RNAs [3]. Following the development and utilization of “Next-generation” sequencing technology and updating bioinformatics technology, circRNAs have been found to play critical roles in cancer growth, metastasis, stemness and resistance to therapy [4–7]. Accumulation of evidences showed that aberrant circular RNA expression has been involved in the pathogenesis and progression of CRC [8–10]. However, the key deregulated circRNAs and their functions in the specific biological processes of tumor progression are still not well-understood.

Hypoxia is one of basic characteristics of the microenvironment in the majority of solid tumors and plays a vital role in cancer cell proliferation, angiogenesis, therapy resistance and development of aggressive tumor phenotype [11–14]. Hypoxia inducible factor 1α (HIF-1α) is a master gene in hypoxia-induced microenvironment, which controls the expressions of oncogene transcription and other relevant target genes that participate in tumor cell proliferation, invasion and metastasis [15–18]. However, there are few studies on the role of specific circRNAs in regulating hypoxia-induced CRC metastasis, and the circRNAs that are relevant to regulate HIF-1α levels in CRC cells remain largely unknown.

In this study, we investigated circ-ERBIN, a circRNA derived from exons 2 to 4 of the ERBIN (ERBB2 interacting protein, also known as ERBB2IP) gene. We demonstrated here that circ-ERBIN promotes the proliferation, invasion, angiogenesis and metastasis of CRC by targeting miR-125a-5p and miR-138-5p and thus synergistically increases the expression of the eIF4E-binding protein 1 (4EBP-1), which subsequently enhances HIF-1α protein expression and activation of HIF-1α pathway. Most importantly, our study suggests that circ-ERBIN is a novel player in CRC progression by hijacking HIF-1α pathway.

## Materials and methods

### Human tissue specimens

Fifty-nine samples of CRC and paired adjacent colorectal tissues were collected with the consent of patients and the experiments were approved by ethics committee of Affiliated Hospital of Jiangnan University, Wuxi, China. All tissue specimens were stored at − 80 °C until use.

### Cell lines and cell cultures

HCT116, SW480, RKO, LoVo, DLD-1, HT-29, HEK293 cells were purchased from the Cell Bank of the Chinese Academy of Sciences (Shanghai, China) and HEK293 cells were purchased from ATCC. The cells were all cultured as described recently [9].

### Actinomycin D assay

HCT116 and RKO cells were equally seeded in 5 wells in 6-well plates (5 × 10^4^ cells per well). Overnight, the cells were exposed to actinomycin D (2 μg/ml, Abcam, ab141058) for 0 h, 4 h, 8 h, 12 h and 24 h, respectively. The cells were then harvested and the mRNA levels of liner or circular form of ERBIN gene were analyzed by qRT-PCR and normalized to the values measured in the mock treatment group (the 0 h group).

### RNA isolation and qRT-PCR

The nuclear and cytoplasmic fractions were extracted using Nuclear and Cytoplasmic Protein Extraction Kit (Beyotime, P0028). Total RNA was isolated using TRIzol as described before [9, 19]. Total RNA(1 μg) was incubated 30 min at 37°Cwith or without 3 U/μg of RNase R (Epicentre Technologies) to verify the existence of circRNA as described before [9]. For mRNA and circRNA, cDNA was synthesized with the PrimeScript RT Master Mix (Takara, Dalian, China) from 1 μg of RNA. The divergent primers were used to determine the abundance of circRNA. Expression levels normalized to the expression of GAPDH mRNA. The expression of miRNAs was detected using Bulge-loop™ miRNA qRT-PCR Primers Sets (one RT primer and a pair of qPCR primers for each set), designed by RiboBio (Guangzhou, China). U6 acted as normalized controls. qRT-PCR was performed according to the manufacturer’s instructions and the relative fold-change was calculated by the 2-△△Ct method. Primers used for real-time reactions are listed in Additional file 2: Table S1. All experiments were repeated at least three times.

### Transwell assay

In brief, 200 μL cells (2×10^5^ cells) resuspended with free medium, were added to the upper compartment of migration or invasion chambers (BD Biosciences). The bottom chamber was filled with 500 μL cell culture medium with 10% FBS as an attractant. After 24 h, cells were fixed and stained with Wright-Giemsa and counted at ×200 magnification in 10 randomly chosen fields. Experiments were repeated three times.

### Plasmids, siRNAs and stable cell lines construction

The sequence of circ-ERBIN was amplified and cloned into a circRNA overexpression vector pLCDH-ciR (Geneseed, Guangzhou, China). Through restriction enzyme sites EcoRI and BamHI, and also confirmed by sequencing. Primers used for cloning were listed in Additional file 2: Table S1. Three siRNAs targeting circ-ERBIN were designed and synthesized by Geneseed (Guangzhou, China). The sequences of circ-ERBIN siRNAs were listed in Additional file 2: Table S1. HCT116 and RKO cells were incubated with circ-ERBIN overexpression or siRNA lentivirus for 48 h and followed by puromycin (5μg/ml) selection for 4 weeks.

### Luciferase reporter assay

The luciferase reporter vector including full length of 4EBP-1 3′ untranslated region (3’UTR) or circ-ERBIN sequences were constructed through gene synthesis procedure. The mutant luciferase reporter vectors were also generated by gene synthesis, respectively. The dicistronic reporter plasmids were constructed by VectorBuilder(Yunzhou Biotechnology (Guangzhou) Co., Ltd). Simply, the plasmid pRnegF is derived from pGL3(Promega), with the Renilla luciferase and firefly luciferase and separated by a short linker sequence. The plasmid pRhif-1αF contains the HIF-1α 5′-UTR sequence that was inserted into the linking region between the Renilla and firefly luciferase coding regions, as firefly luciferase is driven from the putative IRES. Transient transfections were performed with Lipofectamine 2000 (Invitrogen) and Lipofectamine RNAiMIX following the manufacture’s protocol. Cells were lysed 24-48 h after transfection and fluorescence intensity was assayed using a luciferase reporter assay system (Promega). Each transfection also included β-gal for normal control.

### Western blot

Whole cell protein extractions were performed as previously described [19]. Western blots analyses were performed with commercially available antibodies, 4EBP-1 (Cell signaling technology, #9644), HIF-1α (Abcam, ab51608) and Tubulin (Cell signaling technology, #2144S). Phospho-4EBP-1 (Thr37/46), non-phospho-4EBP-1 (Thr37/46), Phospho-4EBP-1 (Ser65) and Phospho-4EBP-1 (Thr70) were purchased as 4E-BP antibody sampler kit (Cell signaling technology, #9955). All experiments were repeated at least three times.

### In vivo experiments

To monitor the tumor growth, 1×10^6^ cells were subcutaneously injected into nude mice (BALB/c, SPF grade, 3–5 weeks old, *n* = 3 per group). Moreover, we also performed experiments that directly injected AgomiR-125a-5p/AgomiR-138-5p into implanted tumor at the dose of 10 nmol (in 40 μl PBS) per mouse every 10 days for two times. Then 4 weeks later, mice were killed and tumors were removed for qRT-PCR, western blot, HE staining and IHC experiments. Tumor volume (V) was monitored by measuring the length (L) and width (W) with vernier caliper and calculated with the formula V = (L × W^2^) × 0.5.

To evaluate in vivo metastasis, HCT116 stable cells as described in the article were injected into the lateral vein in the nude mouse tail (BALB/c, SPF grade, 4–6 weeks old, *n* = 10 per group). After 8 weeks, mice of each group were sacrificed. Lung tissues were collected for metastatic foci evaluation and standard histopathological study. All manipulations involving live mice were approved by the Animal Care and Use Committee of Soochow University.

### Immunohistochemistry

Immunohistochemistry was performed according to a standard protocol as described before [20]. Primary antibodies of anti-HIF-1α (Abcam, ab51608), anti-4EBP-1 (Proteintech, 60,246–1-lg) and anti-CD31 (Abcam, ab76533) were used. Negative controls were performed by replacing the primary antibody with PBS. The slides were analyzed separately by two independent observers. The staining intensity was scored on a scale of 0–3 as negative (0), weak (1), medium (2) or strong (3). The extent of the staining, defined as the percentage of positive staining areas of tumor cells in relation to the whole tumor area, was scored on a scale of 0 (0%), 1 (1–25%), 2 (26–50%), 3 (51–75%) and 4 (76–100%). An overall protein expression score (0–12) was calculated by multiplying the intensity and extent positively scores.

### Statistical analysis

The Student’s t-test or one-way ANOVA was used to detect the difference of circ-ERBIN, miR-125a-5p, miR-138-5p and 4EBP-1 expression between different groups. The results are presented as the mean ± standard deviation. Statistical significance was determined by Student two-tailed *t* test. *P-*value <0.05 was considered significant and marked with *. Each experiment was performed in at least three independent experiments.

## Results

### Identification of circ-ERBIN as a candidate gene for metastasis of CRC

ERBIN, ERBB2-interacting protein (ERBB2IP), stabilizes ERBB2 at the plasma membrane through its PDZ interaction [21]. Several studies including ours have demonstrated that Erbin is involved in CRC [20], hepatocellular carcinoma (HCC) [22], breast cancer [23].

**Fig. 1:**
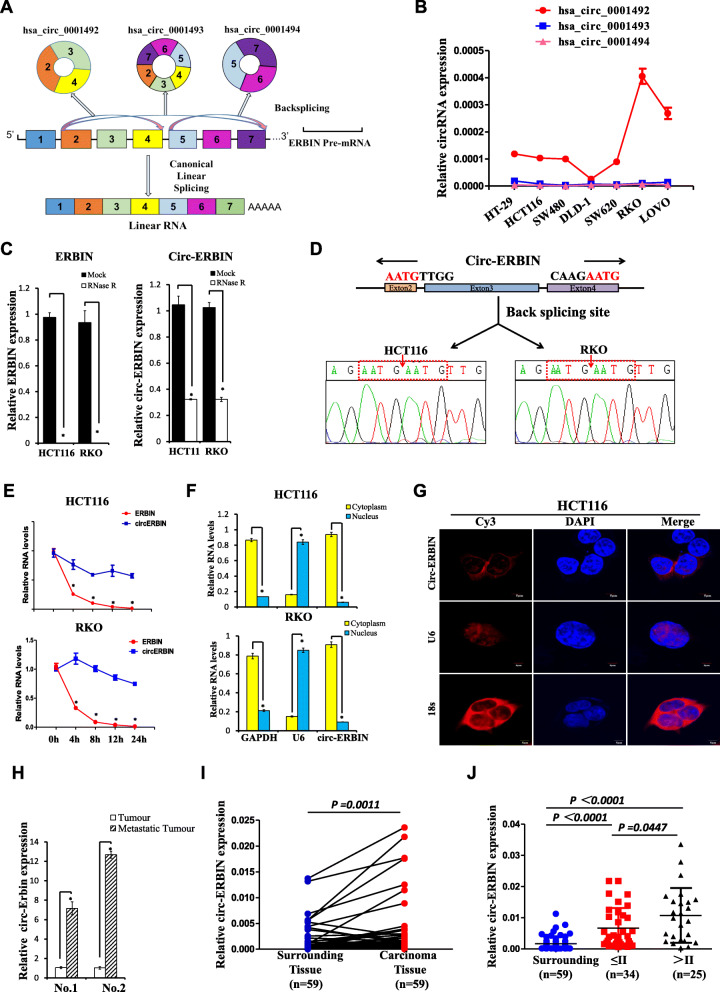
Identification of circ-ERBIN as a candidate gene for metastasis of CRC. **a** Schematic illustration of three circRNAs in *ERBIN* gene. **b** RNA expression of the three circRNAs in different CRC cell lines was detected using qRT-PCR. **c**, **d** qRT–PCR for the abundance of *ERBIN* (left) and circ-ERBIN (right) RNA levels in HCT116 and RKO cells treated with RNase R (**c**) and followed by Sanger sequencing (**d**). The amount of *ERBIN* and circ-ERBIN RNA were normalized to the value measured in the mock treatment. **e** The relative RNA levels of *ERBIN* and circ-ERBIN were analyzed by qRT-PCR after treatment with Actinomycin D at the indicated time points in HCT116 cells. **f**, **g** qRT-PCR (**f**) and FISH (g) data indicating the circ-ERBIN is abundant in the cytoplasm. **f** *GAPDH* and *U6* were applied as positive controls in the cytoplasm and nucleus for qRT-PCR experiments, respectively. **g** 18 s and U6 were applied as positive controls in the cytoplasm and nucleus for Fluorescence in situ hybridization (FISH) experiments, respectively. Nuclei were stained with DAPI. **h** qRT–PCR for the abundance of circ-ERBIN RNA in the samples of a mouse model of CRC metastasis. **i** RNA levels of circ-ERBIN in 59 pairs randomly selected CRC samples were detected by the junction primers (*P* < 0.001). **j** Expression of circ-ERBIN in different stages of CRC carcinoma samples. Values are the average of three independent experiments. Data are expressed as the mean ± SD, *P*<0.001

According to the annotation of circBase (http:// www.circbase.org/), there are 3 different splicing products on the human ERBIN pre-mRNA sequence (Fig. 1a). Interestingly, qRT-PCR experiments showed that only has_circ_0001492, termed as circ-ERBIN, which is formed by circularization of 2–4 exons of the ERBIN gene, was highly expressed in different CRC cells, and significantly increased in more aggressive CRC cells (Fig. 1b). CircRNAs have been reported to be conserved across different species [2], we further analyzed the difference on the sequence of circ-ERBIN between human and mouse. As shown in Additional file 1: Figure S1A, they were 94% homologous. Moreover, circ-Erbin was circularized by the exon 2–4 of Erbin gene, indicating the evolutionary conservation of circ-ERBIN. To confirm the existence of circ-ERBIN, HCT116, RKO and tumor tissue from mice were treated with RNase R, a processive 3′ to 5′ exoribonuclease [24], respectively. qRT-PCR results showed circ-ERBIN had a stronger resistibility to RNase R, while linear form of ERBIN was digested sharply (Fig. 1c and Additional file 1: Figure S1B). Additionally, Sanger sequencing affirmed the junction sites (Fig. 1d and Additional file 1: Figure S1C). Moreover, actinomycin D [25] treatment showed that circ-ERBIN is more stable than linear ERBIN in HCT116 and RKO cells (Fig. 1e). Furthermore, qRT-PCR experiments of nuclear mass separation and Fluorescence in situ hybridization (FISH)) demonstrated that circ-ERBIN was predominantly localized in the cytoplasm (Fig. 1f and g, Additional file 1: Figure S1D). Notably, when we treated HCT116 and RKO cells with serum-free media (SFM) (Additional file 1: Figure S1E), Cobalt chloride (Cocl2, a hypoxia mimetic) (Additional file 1: Figure S1F) or TGF-β(Additional file 1: Figure S1G), a key factor involved in cancer progression and metastasis, we found that both hypoxia and TGF-β stimulation promoted expressions of circ-ERBIN, suggesting that circ-ERBIN might be a circular RNA associated with cancer progression.

To identify essential circRNAs in CRC metastasis, we established TRAI mouse model, a mouse model of CRC liver metastasis by a trans-anal injection of murine CMT-93 submucosally into the distal and posterior rectum of C57BL/6 J mice, for CRC liver metastasis. We studied the differential expressed circRNAs between liver metastases and primary CRC tumors in TRAI mouse models by RNA-seq analysis as described before [9]. Confirmed by qRT-PCR experiments, circ-Erbin expression was higher in liver metastases than in its paired primary tumors (Fig. 1h). We also found that the expression of circ-ERBIN was higher in CRC tissues compared to their counterpart surrounding tissues (Fig. 1i).Notably, circ-ERBIN was expressed more highly in stage III/IV tissues than in stage I/II samples (Fig. 1j). Collectively, these data demonstrates that circ-ERBIN is a novel candidate gene for CRC metastasis.

### Overexpression of circ-ERBIN accelerates proliferation, migration, invasion and metastasis of CRC cells in vitro and in vivo

**Fig. 2:**
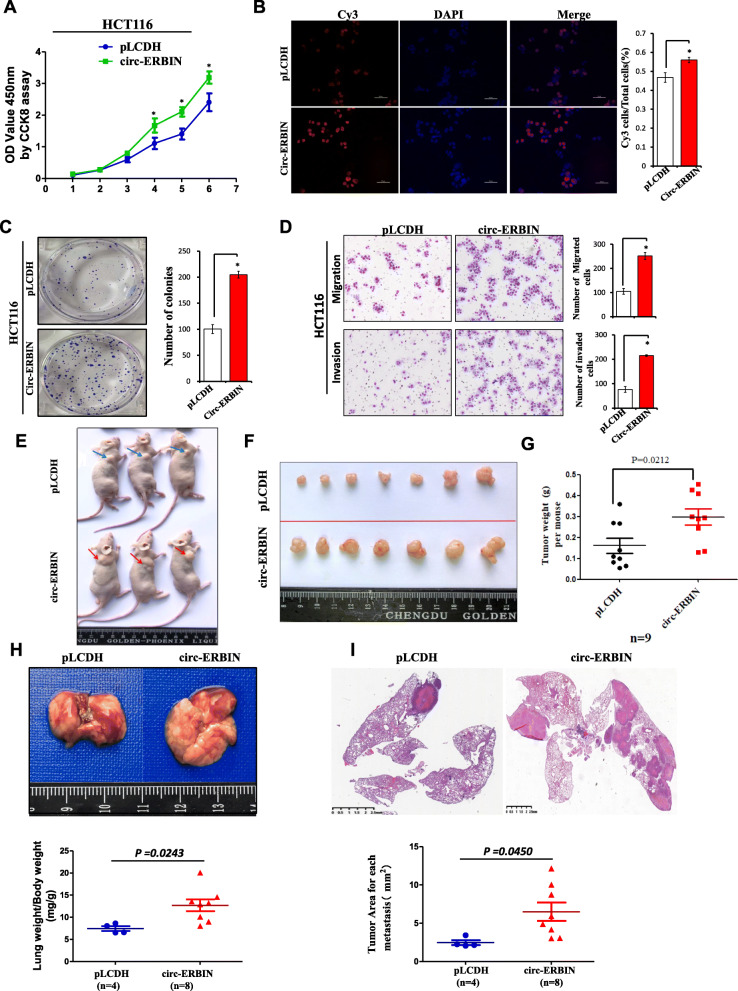
Overexpression of circ-ERBIN promotes proliferation, migration, invasion of CRC in vitro and in vivo. **a**-**c** CCK8, EdU and colony formation assays in circ-ERBIN op stable cells and its control pLCDH cells. **d** Cell migration and invasion assays were performed and measured in circ-ERBIN op stable cells and its control pLCDH cells. Data are means ± SD. *, *P*<0.05 vs pLCDH. **e**-**g** HCT116 stably expressed circ-ERBIN or pLCDH vector were injected into BALB/C nude mice. Tumor volumes and weights were analyzed. **h**-**i** HCT116 cells (2 × 10^6^ cells/mouse) with circ-ERBIN overexpression were injected through tail vein into nude mice to produce lung metastasis model of CRC. **h** Eight weeks later, mice were sacrificed and lungs were isolated (upper panel). Lung weight/Body weight value (mg/g) was calculated (bottom panel). **i** Images of H&E staining are shown and tumor area was measured

Gain-of-function assays were performed to evaluate the effects of circ-ERBIN overexpression on the biological behaviors of CRC cells. A circRNA expression vector, as described before [26], was used to over-express circ-ERBIN in HCT116 cells. Firstly, we transfected pLO-circ-ERBIN plasmids into 293 T and HCT116 cells, and Sanger sequencing and qRT-PCR analysis showed the circularization sites and expression efficiency of designed construct and confirmed that the transfection had no influence on the parental gene of circ-ERBIN (Additional file 1: Figure S2A-S2C). Next, we overexpressed pLO-circ-ERBIN plasmids into HCT116 cells for further study. The results showed that circ-ERBIN significantly up-regulated the migration of HCT116 cells (Additional file 1: Figure S2D). To further validate the effects of circ-ERBIN overexpression on CRC cells, we constructed circ-ERBIN stable overexpression cell lines by using lentivirus-GFP-circ-ERBIN (circ-ERBIN op) and its negative control (pLCDH), respectively. Fluorescent imaging and qRT-PCR analyses revealed that circ-ERBIN was greatly increased in HCT116-circ-ERBIN stable cells compared to those in the control cells (Additional file 1: Figure S2E). Then, we performed the CCK8, EdU and colony formation assays to analyze the effect of circ-ERBIN on cell proliferation. As shown, circ-ERBIN op cells showed significantly higher cell proliferation, EdU incorporation and colony formation (Fig. 2a-c) than control cells. Moreover, transwell migration and invasion assays showed the increased ability of circ-ERBIN op cells compared to pLCDH cells (Fig. 2d).

To further investigate the effects of circ-ERBIN overexpression in vivo, mouse models of xenograft tumor growth and lung metastasis were performed. As indicated, circ-ERBIN overexpression significantly promoted tumor growth (Fig. 2e-g) in vivo. Moreover, in xenograft lung metastasis of mouse models by injecting circ-ERBIN op or pLCDH cells into nude mice through tail vein, the value of Lung weight/Body weight (Fig. 2h) and HE staining data (Fig. 2i) showed that circ-ERBIN op cells developed more lung metastases 8 weeks later after administration. Collectively, these results demonstrate that circ-ERBIN overexpression promotes CRC tumor growth and progression.

### Knockdown of circ-ERBIN represses CRC cell proliferation, migration and invasion

**Fig. 3:**
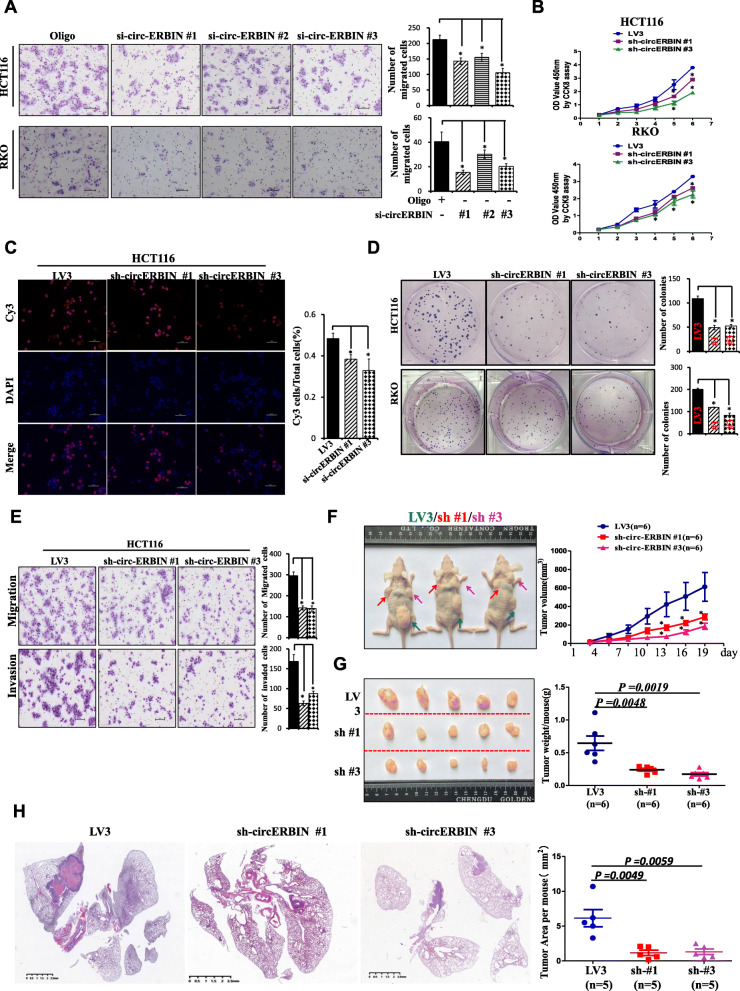
Circ-ERBIN knockdown suppresses CRC cells proliferation, migration and invasion in vitro and in vivo. **a** Transwell migration assays in HCT116 and RKO cells after circ-ERBIN siRNAs transfection. **b**-**d** CCK8, EdU and colony formation assays in HCT116 cells after circ-ERBIN knockdown. **e** HCT116 sh-circ-ERBIN stable cells were analyzed for their in vitro migration and invasion using transwell chambers and invasion chambers, respectively. **f**-**g** HCT116 sh-circ-ERBIN stable cells were injected into BALB/C nude mice (*n* = 6 for each group). Tumor volumes and weights were monitored. **h** The number of metastases in mice injected with sh-circ-ERBIN #1/#3 stable cells through tail vein into nude mice to produce lung metastasis model of CRC. Images of H&E staining (Left) are shown and tumor area was measured (Right). Data are means ± SD. * and #, *P*<0.05 vs LV3 or Oligo

To further test the functions of circ-ERBIN in CRC cells, three siRNAs that targeted the junction sites of circ-ERBIN were designed and transfected into HCT116 and RKO cells. Circ-ERBIN expression was greatly knocked down by si-circ-ERBIN #1 and si-circ-ERBIN #3, while both of them had no effect on the level of linear mRNA sequence (Additional file 1: Figure S3A). Transwell migration assay showed that si-circ-ERBIN siRNAs transfection significantly suppressed CRC cells (HCT116 and RKO) migration (Fig. 3a). Furthermore, we established circ-ERBIN-knockdown stable cell lines in HCT116 and RKO using lentivirus packaged circ-ERBIN shRNAs (sh-circ-ERBIN #1 or #3) for CCK8, EdU and colony formation assays. The efficiency of circ-ERBIN deletion by qRT-PCR assays was confirmed (Additional file 1: Figure S3B). Compared to the negative control (LV3) group, proliferative abilities of HCT116 and RKO were inhibited in the sh-circ-ERBIN cells (Fig. 3b-d and Additional file 1: Figure S3C). Additionally, trans-well migration and invasion assays revealed that the number of cells was fewer in the knockdown stable cells than that in the LV3 group (Fig. 3e and Additional file 1: Figure S3D). To investigate the effects of circ-ERBIN knockdown on tumor growth and metastasis of CRC in vivo, mouse models of xenograft tumor growth and lung metastasis were performed. Our results showed that circ-ERBIN knockdown significantly suppressed tumor growth (Fig. 3f and g) and lung metastasis of CRC in vivo (Fig. 3h and Additional file 1: Figure S3E). Taken together, these findings demonstrated that circ-ERBIN knockdown inhibited the progression of CRC in vitro and in vivo.

### Circ-ERBIN increases tumor angiogenesis and HIF-1α protein levels in CRC

**Fig. 4:**
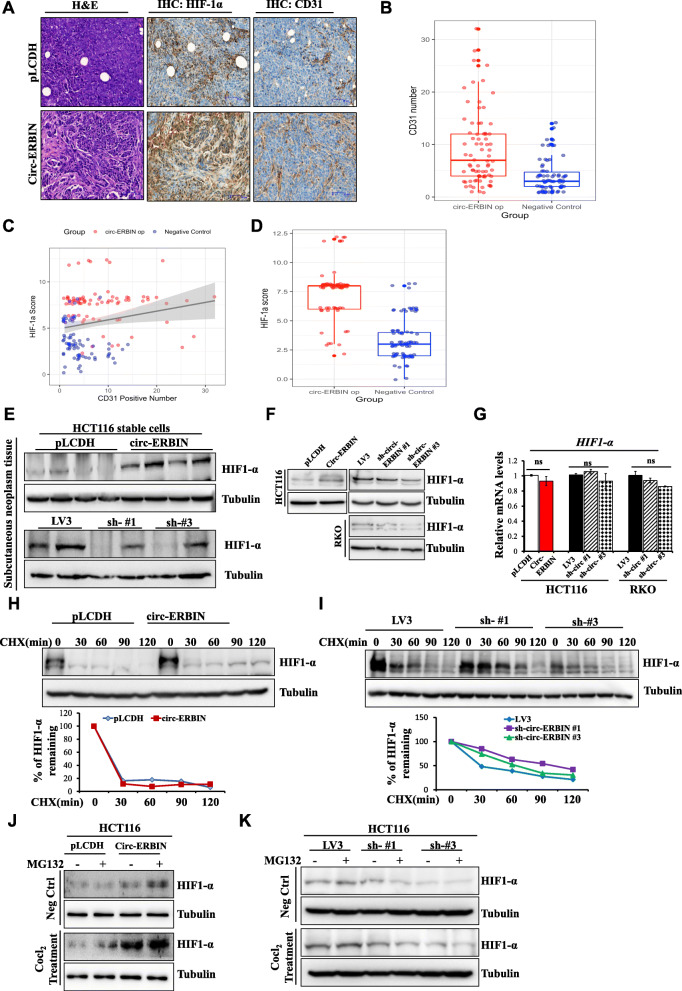
Circ-ERBIN exerts an oncogenic role via alleviating HIF-1α levels in CRC cells. **a** HE staining and IHC experiments for CD31 and HIF-1α in subcutaneous tumors formed by circ-ERBIN op and pLCDH cells. **b** Microvascular densities (MVD) of the indicated xenograft tumors were detected by CD31 staining. Scale bars: 100 μm. *P*<0.001. **c** Correlation between CD31 positive number and HIF-1α expression. R = 0.198, *P* = 0.015. **d** IHC score for HIF-1α in the indicated subcutaneous tumors. *P*<0.001. **e** Western blot experiments were performed using subcutaneous tumor tissues formed by circ-ERBIN op or sh-circ-ERBIN cells, respective antibodies were used. **f**, **g** Western blot (**f**) and qRT-PCR (**g**) experiments were performed using stable overexpression or knockdown of circ-ERBIN cell lines. **h**, **i** Circ-ERBIN op (**h**) or knockdown (**i**) cells were treated with Cocl_2_ for 24 h and followed by CHX treatment for different minutes. Western blot experiments were performed to test the degradation rate of HIF-1α. **j**, **k** Circ-ERBIN op (**j**) or knockdown (**k**) cells were treated with Cocl2 for 24 h and followed by MG132 treatment for 4 h

Tumor angiogenesis is an important process for tumor growth and metastasis and is usually related to hypoxia. Interestingly, we observed that tumors formed by circ-ERBIN op stable cells grew larger and more quick, suggesting tumor angiogenesis and HIF-1α signaling were up-regulated in these tumors. Therefore, the number of microvessels by staining CD31 (also called PECAM-1), a sensitive vascular marker, were analyzed to evaluate tumor angiogenesis in tumors. Significantly, the number of microvessels detected by CD31 staining was greatly increased in circ-ERBIN op tumors (Fig. 4a and b) compared with that in control tumors. As we have known, hypoxia plays a pivotal role in cancer through the alteration of microenvironment and promoting formation of blood vessels, and hypoxia inducible factor (HIF-1α) is a master gene in mediating different hypoxia related cell processes, including tumor angiogenesis [27–29]. IHC staining showed significantly increased expression of HIF-1α in circ-ERBIN op subcutaneous tumors (Fig. 4a and d) and lung metastatic tumors (Additional file 1: Figure S4A) compared with that in control tumors. Moreover, there was a positive correlation between number of CD31 positive microvessels and HIF-1α staining of tumors (Fig. 4c). Meanwhile, circ-ERBIN knockdown significantly decreased HIF-1α expression in subcutaneous tumors and lung metastatic tumors (Additional file 1: Figure S4A).

To further confirm the hypothesis that circ-ERBIN up-regulates HIF-1α expression in vivo, we analyzed the expression levels of HIF-1α mRNA and protein in the subcutaneous tumors from mouse models by western blot and qRT-PCR experiments. Circ-ERBIN up-regulation increased while circ-ERBIN down-regulation decreased expression levels of HIF-1α protein but not HIF-1α mRNA respectively (Fig. 4e and Additional file 1: Figure S4B and S4C). The results were similar in our in vitro experiments (Fig. 4f, g and Additional file 1: Figure S4D). Notably, hypoxia could exacerbate the effects of circ-ERBIN on HIF-1α protein levels but not HIF-1α mRNA levels when the cells were treated with Cobalt chloride (Cocl2), a hypoxia mimetic (Additional file 1: Figure S4E and S4F).

To further test the hypothesis that circ-ERBIN regulates HIF-1α expression in the translational levels, circ-ERBIN op or knockdown stable cells were firstly treated with Cocl2 for 24 h and then followed by CHX treatment at varying time points, the expression levels of HIF-1α were determined. As shown in Fig. 4h and i, similar HIF-1α degradation rates were observed regardless of circ-ERBIN levels in cells, suggesting that degradation of HIF-1α per se was unlikely affected by circ-ERBIN. To exclude the possibility that circ-ERBIN affects HIF-1α stability through proteasome-dependent degradation, MG132, the proteasome inhibitor exhibited sustained enhancement or no obvious rescue of the increased or decreased HIF-1α protein levels in the circ-ERBIN op or sh-circ-ERBIN cells, respectively (Fig. 4j and k). Altogether, we found that circ-ERBIN increases HIF-1α protein levels independent of degradation or post-translational modifications of HIF-1α.

### Circ-ERBIN elevates the expression of HIF-1α via 4EBP-1

**Fig. 5:**
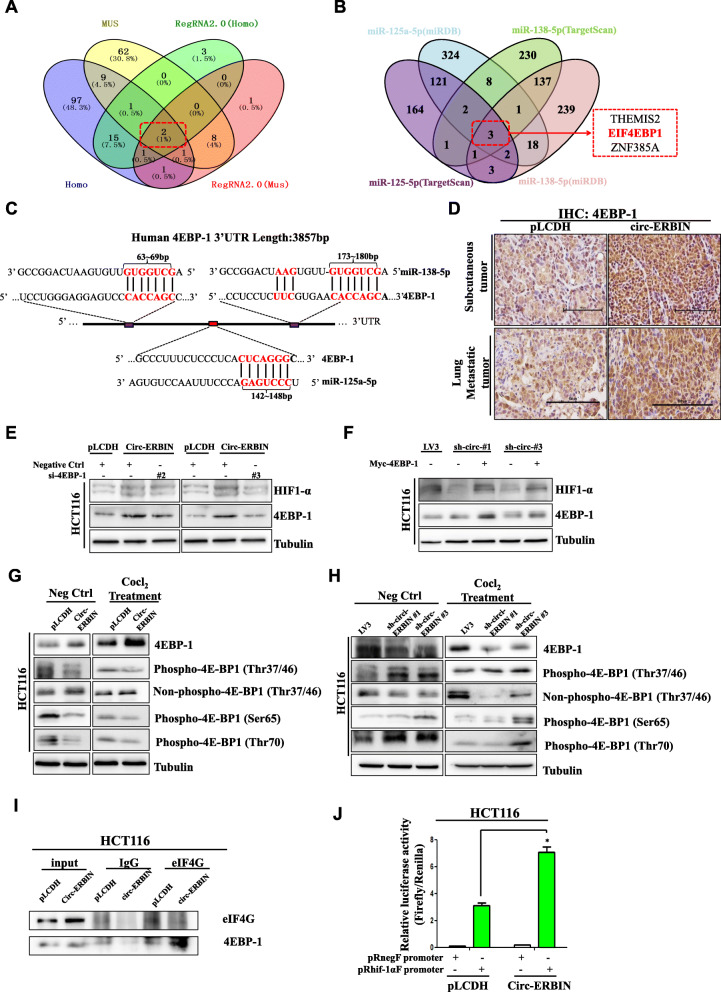
Circ-ERBIN elevates the expression of HIF-1α via 4EBP-1*.* **a** Venn diagram for the putative target miRNAs of circ-ERBIN. **b** Venn diagram for the downstream targets of circ-ERBIN targeted miRNAs. **c** Schematic diagram of the predicted miR-125a-5p and miR-138-5p binding sites for 4EBP-1. **d** Circ-ERBIN overexpression (pLCDH/circ-ERBIN) stable cells were used to perform subcutaneous tumor model and tail vein injection metastasis model, respectively. Immunohistochemical staining of 4EBP-1 were presented. Scale bars, 100 μm. **e**, **f** 4EBP-1 siRNA or 4EBP-1 plasmids were transfected into HCT116 circ-ERBIN op or knockdown cells, respectively. Western blots were performed to determine the corresponding proteins. **g**, **h** Western blot experiments were performed using HCT116 circ-ERBIN stable overexpression or knockdown cell lines, antibodies with different phosphorylation sites of 4EBP-1 were used respectively. **i** Circ-ERBIN op or pLCDH cells were immunopreipitated (IP) with IgG or eIF4G antibody, and the immunoprecipitates were analyzed by western blot. **j** Circ-ERBIN op or pLCDH cells were transiently transfected with pRnegR or pRhif-1αF plasmids. Renilla and firefly luciferase activities were determined after 24 h tranfection, and IRES activities represented as ratios of Firefly to Renilla luciferase. Data are means ± SD. *, *P*<0.05.

To find the mechanism involved in circ-ERBIN mediated HIF-1α protein expression, we used miRanda and RegRNA2.0 prediction tool to predict the target miRNAs and selected 2 miRNA candidates (miR-125a-5p, miR-138-5p) that may be the targets of circ-ERBIN by intersection analysis (Fig. 5a). Nevertheless, HIF-1α is not the remarkable target of either miR-125a-5p or miR-138-5p. Interestingly, we found that eukaryotic translation initiation factor 4E (eIF4E)-binding protein 1 (4EBP-1), a protein which plays important roles in cap-independent HIF1-α protein translation in hypoxic microenvironment [30], is both the target of miR-125a-5p and miR-138-5p (Fig. 5b). Moreover, we identified three conserved binding sites for miR-125a-5p and miR-138-5p on the 3′ UTR region of 4EBP-1 (Fig. 5c). To test the hypothesis that circ-ERBIN may regulate HIF-1α expression through 4EBP-1, we first analyzed the protein levels of 4EBP-1 in subcutaneous tumors formed by circ-ERBIN stable cells, and found that the levels of 4EBP-1 positively correlated with the levels of circ-ERBIN (Additional file 1: Figure S5A). Similar results were found in our IHC experiments (Fig. 5d and Additional file 1: Figure S5B). To further studied whether the effects of circ-ERBIN on HIF-1α are dependent on 4EBP-1, we transfected siRNAs targeting 4EBP-1, as confirmed by western blot in Additional file 1: Figure S5C, into circ-ERBIN op stable cells, the data indicated that knockdown of 4EBP-1 significantly reversed the enhancing effects of circ-ERBIN overexpression on HIF-1α protein levels in circ-ERBIN op cells (Fig. 5e). Furthermore, in sh-circ-ERIBN stable cells, 4EBP-1 overexpression significantly attenuated the inhibitory effects of circ-ERBIN knockdown on HIF-1α protein levels (Fig. 5f). Notably, in circ-ERBIN op stable cells with or without Cocl_2_ treatment, we found that 4EBP-1 protein levels were significantly increased but phosphorylated 4EBP-1 at several conserved sites were all significantly decreased (Fig. 5g). Accordingly, in circ-ERBIN knockdown stable cells, 4EBP-1 protein levels were significantly decreased but phosphorylated 4EBP-1 at several conserved sites were all significantly increased (Fig. 5h). Moreover, immunoprecipitation (IP) experiments indicated that binding capacity between eIF4G and 4EBP-1 was greatly increased in circ-ERBIN overexpression cells (Fig. 5i), whereas the expression of eIF4G was not changed in circ-ERBIN stable overexpression cells (Additional file 1: Figure S5D). In addition, we constructed pRnegF and pRhif-1αF plasmids to test the cellular HIF-1α IRES efficiency in cells. We found that pRhif-1αF increased the expression of downstream Firefly luciferase relative to Renilla luciferase, and circ-ERBIN overexpression significantly enhanced the effects of pRhif-1αF (Fig. 5j). Altogether, these data indicated that circ-ERBIN overexpression helps to maintain the non-active status of 4EBP-1, and thus leads to a switch from cap-dependent to cap-independent protein translation process in CRC cells.

We further determined whether the effects of circ-ERBIN on CRC progression are related to upregulation of 4EBP-1. Transwell migration assay showed that 4EBP-1 siRNAs successfully reversed the promoting effect of circ-ERBIN on cell migration (Additional file 1: Figure S5E). At the meantime, 4EBP-1 overexpression also greatly eliminated the inhibitory effect of sh-circ-ERBIN on cell migration in HCT116 cells (Additional file 1: Figure S5F). Generally, circ-ERBIN promotes CRC progression via 4EBP-1 mediated cap-independent HIF-1α translation functionally.

### Circ-ERBIN elevates HIF-1α expression through miR-125a-5p/miR-138-5p/4EBP-1 signaling

**Fig. 6:**
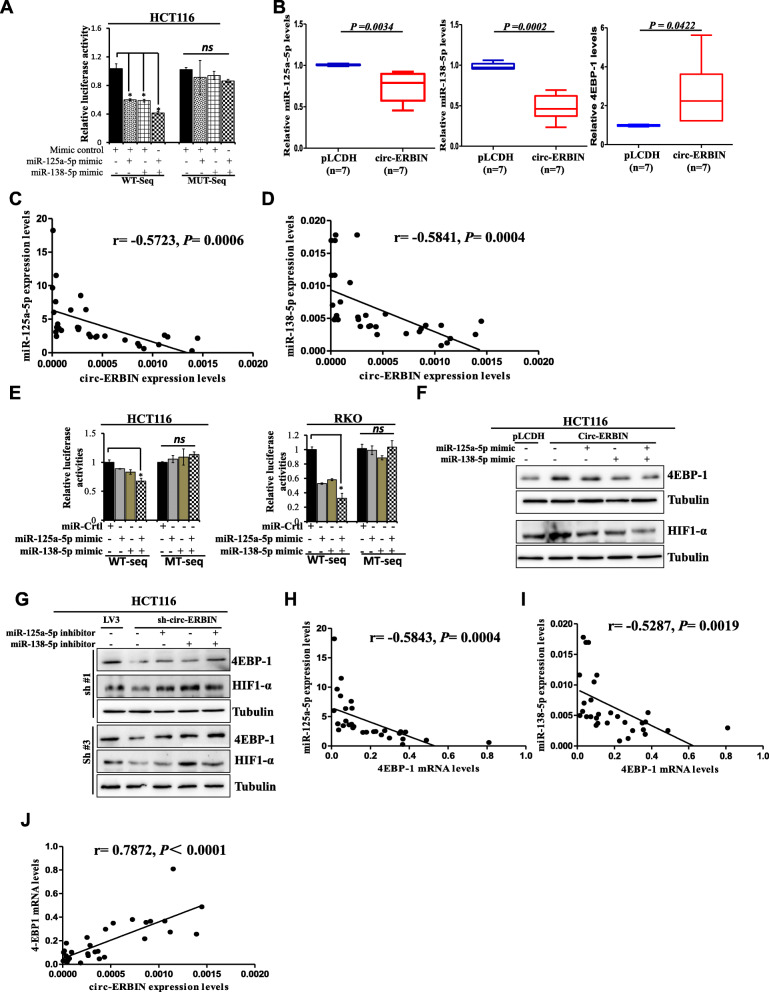
Circ-ERBIN elevates HIF-1 α expression by miR-125a-5p/miR-138-5p/4EBP-1 signaling. **a** Luciferase activity of LUC-circ-ERBIN WT or LUC-circ-ERBIN Mutant in HCT116 cells after co-transfection with miR-125a-5p or/and miR-138-5p mimics. **b** qRT-PCR was performed to detect related miRNAs and 4EBP-1 levels using mouse subcutaneous tumor samples. **c**, **d** qRT-PCR experiments were performed to detect miR-125a-5p and miR-138-5p mRNA levels using clinical CRC patient samples (*n* = 32). The correlations between circ-ERBIN and miR-125a-5p or miR-138-5p were represented. All the qRT-PCR experiments were normalized using GAPDH as an endogenous control. Data are means ± SD. *P*<0.05. **e** Luciferase activity of 4EBP-1 3’UTR constructs harboring wide type sequence or mutant sequence were analyzed after being transfected with miR-125a-5p or miR-138-5p mimics alone or together. β-gal was transfected as control. **f** miR-125a-5p and miR-138-5p mimics were transfected into circ-ERBIN stable cell lines alone or together. Western blots were performed to analyze the protein of 4EBP-1 and HIF-1α. **g** miR-125a-5p or miR-138-5p inhibitor was transfected alone or together into HCT116 sh-circ-ERBIN stable cell lines. Western blots were performed to analyze the protein levels of 4EBP-11 and HIF-1α. **h**, **i** The correlations between 4EBP-1 and miR-125a-5p or miR-138-5p were represented. **j** The correlation between circ-ERBIN and 4EBP-1 was represented. All the qRT-PCR experiments were normalized using GAPDH as an endogenous control. Data are means ± SD. *P*<0.05.

We have bioinformatically predicted miR-125a-5p and miR-138-5p as the targets of circ-ERBIN. Therefore, we further studied the relationship between circ-ERBIN and miR-125a-5p and miR-138-5p. Luciferase reporter plasmids with a wild type sequence of circ-ERBIN (WT-seq) or mutant sequence (MUT-seq) in the binds sites of miR-125a-5p and miR-138-5p were generated, respectively. As shown in the figures, miR-125a-5p or miR-138-5p mimics transfection suppressed the luciferase activity of WT sequence, and co-transfection of both mimics showed best synergistically effects on the luciferase activity of WT plasmids. Meanwhile, the suppressive effects of miR-125a-5p or/and miR-138-5p mimics transfections were significantly abolished in mutant plasmids (Fig. 6a and Additional file 1: Figure S6A). Furthermore, qRT-PCR results showed that circ-ERBIN expressions were negatively correlated with the miR-125a-5p and miR-138-5p in CRC stable cell lines (Additional file 1: Figure S6B and S6C), samples from subcutaneous tumors from xenograft mouse models of CRC (Fig. 6b and Additional file 1: Figure S6D) and clinical CRC samples (Fig. 6c and d).

According to TargetScan and microRNA.org, the web-based prediction software for targets of miRNAs, 4EBP-1 3’UTR region contains the conserved MRE of both miR-125a-5p and miR-138-5p, might be putative target for both miR-125a-5p and miR-138-5p (Fig. 5b). Western blotting showed that transfections of mimics or inhibitors of miR-125a-5p and miR-138-5p significantly reduced or increased 4EBP-1 protein levels, respectively (Additional file 1: Figure S6E and S6F). To evaluate whether 4EBP-1 is the direct target of miR-125a-5p and miR-138-5p, luciferase reporter plasmids with wild type of 4EBP-1 mRNA 3’UTR sequence (WT-seq) and mutant 4EBP-1 mRNA 3’UTR sequence (MUT-seq) were generated. The results revealed that co-transfection of miR-125a-5p and miR-138-5p mimics significantly reduced the luciferase activity of WT-seq, but not that of MUT seq, in HCT116 and RKO cells (Fig. 6e). These results indicated 4EBP-1 was the direct target of both miR-125a-5p and miR-138-5p.

To further confirm the regulation of circ-ERBIN on HIF-1α expression is dependent on miR-125a-5p/miR-138-5p mediated 4EBP-1 levels, we performed the following western blot and qRT-PCR experiments. The data showed a positive correlation between circ-ERBIN and 4EBP-1 in circ-ERBIN op or knockdown stable cells (Additional file 1: Figure S6G) and samples from subcutaneous tumor models (Fig. 6b and Additional file 1: Figure S6D). Importantly, the increased protein and mRNA levels of 4EBP-1 by circ-ERBIN overexpression were dramatically abated by miR-125a-5p and miR-138-5p mimics co-transfection in circ-ERBIN op cells (Fig. 6f) and transient transfection experiments (Additional file 1: Figure S6H). In addition, miR-125a-5p and miR-138-5p inhibitor could partially rescue the effect of circ-ERBIN si/shRNA on the expression of 4EBP-1 (Fig. 6g and Additional file 1: Figure S6I and 6 J). Notably, HIF-1α protein expression had a similar change pattern with that of 4EBP-1 (Fig. 6f and g). Furthermore, we confirmed the negative correlation between 4EBP-1 and miR-125a-5p/miR-138-5p, and positive correlation between 4EBP-1 and circ-ERBIN in human CRC samples (Fig. 6h-j). Taken together, these results indicated that circ-ERBIN increases HIF-1α expression by directly targeting miR-125a-5p/miR-138-5p/4EBP-1 signaling.

### Circ-ERBIN promotes the progression of CRC by activating HIF-1α signaling through the circ-ERBIN/miR-125a-5p/miR-138-5p/4EBP-1 axis

**Fig. 7:**
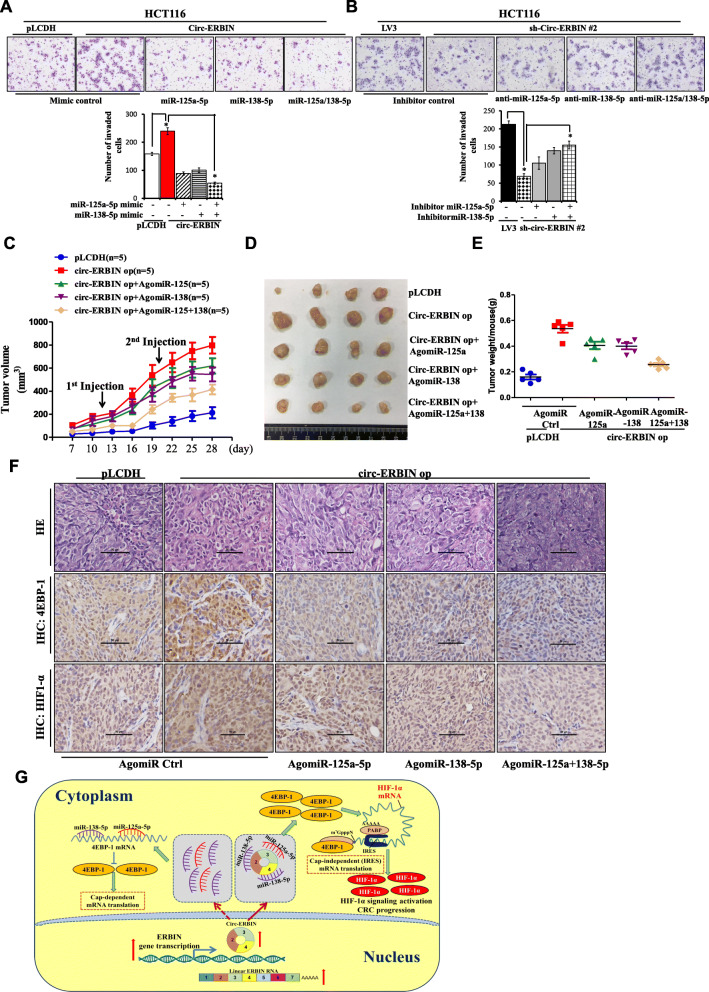
Circ-ERBIN accelerates the growth and metastasis of CRC by activating HIF-1α signaling through the circ-ERBIN/miR-125a-5p/miR-138-5p/ 4EBP-1 pathway. **a** Transwell assays for HCT116 cells that stably overexpressed circ-ERBIN or co-transfected with miR-125a-5p or miR-138-5p mimics after 2 days. **b** MiR-125a-5p or miR-138-5p inhibitor were transfected alone or together into HCT116 sh-circ-ERBIN #1 stable cell lines for 2 days followed by the analysis of cell migration using transwell assays. Cells were stained by Giemsa’s staining and visualized under a phase-contrast microscope. **c**-**e** HCT116 stably expressed circ-ERBIN or pLCDH vector were injected into BALB/C nude mice (*n* = 5 for each group). AgomiRs were injected into tumors as represented. The tumors were excised and photographed, and the size and weight of tumors were presented. **f** Representative immunohistochemical stainings of 4EBP-1 and HIF-1α in subcutaneous tumors with agomiRs injection as described. Scale bars, 100 μm. **g** A schematic model displaying the role of circ-ERBIN/miR-125a-5p/miR-138-5p/ 4EBP-1/HIF-1α axis in CRC metastasis.

Given the roles of circ-ERBIN and 4EBP-1 in CRC progression, we studied the roles of miR-125a-5p and miR-138-5p in circ-ERBIN mediated migration and invasion of colorectal cancer cells. Transwell invasion assay showed that co-transfection of miR-125a-5p and miR-138-5p mimics significantly attenuated the up-regulation of invasion and migration in circ-ERBIN overexpression cells (Fig. 7a and Additional file 1: Figure S7A). In addition, miR-125a-5p and miR-138-5p inhibitors greatly abolished the inhibitory effects of invasion and migration in sh-circ-ERBIN cells (Fig. 7b and Additional file 1: S7B and S7C).

To investigate the functions of circ-ERBIN/miR-125a-5p/miR-138-5p axis in vivo, the stable HCT116 cells with pLCDH or circ-ERBIN op were subcutaneously injected into nude mice as described before. As shown in Fig. 7c, after the tumors were formed, Ago-miR-NC, Ago-miR-125a-5p and Ago-miR-138-5p were injected separately or together into the tumors every 10 days, and tumor growth was measured every 3 days beginning at day 7 until day 28. Tumors injected with both Ago-miRs represented significantly slower growth rate than those with the Ago-miR-NC as control (Fig. 7c). The tumors were excised and photographed, the size and weight of tumors with Ago-miR125a/miR-138-5p injection were smaller than that with Ago-miR-NC (Fig. 7d-e and Additional file 1: Figure S7D). Further validations were performed by qRT-PCR experiments (Additional file 1: Figure S7E and S7F). Notably, the data from qRT-PCR (Additional file 1: Figure S7G), western blot (Additional file 1: Figure S7H) and immunohistochemistry (Fig. 7f) all revealed the existence of circ-ERBIN/miR-125a-5p/miR-138-5p/4EBP-1/HIF-1α axis in vivo. Collectively, these data demonstrates that circ-ERBIN promotes the tumor growth and progression of CRC through the miR-125a-5p/miR-138-5p/4EBP-1axis mediating the activation of HIF-1α pathway.

## Discussion

CircRNAs, owning longer half-lives and stronger stabilities than their linear forms, have been identified from splicing errors to important molecules that play critical roles in the initiation and development of diverse human cancers, including gastric cancer [31], breast cancer [32], lung cancer [33], hepatocellular carcinoma [34] and colorectal cancer [10]. Importantly, circRNAs have been proven to be potential biomarkers in cancer as their tissue-specific and cell-specific patterns [35, 36]. However, the sensitive and specific circRNAs and their molecular mechanisms in CRC remain elusive.

In our research, we firstly identified circ-ERBIN as a novel and up-regulated circRNA that plays important roles in CRC. Circ-ERBIN derives from 2 to 4 exons of Erbin gene, which has a specific and high expression pattern in epithelial cells. Additionally, Erbin has indispensable roles in maintaining cell polarity and accumulating studies have demonstrated that Erbin plays a critical role in cancer [23]. Our recent findings showed that Erbin promotes the tumorigenesis and tumor growth of CRC and the expression of Erbin is elevated in tumor samples from CRC patients [20]. Strikingly, we found circ-ERBIN has a similar expression pattern with Erbin, its host gene, in clinical CRC tissues. Functionally, in vitro and in vivo experiments showed circ-ERBIN up-regulation could promote the proliferation, invasion, growth and metastasis of CRC, whereas the knockdown of circ-ERBIN could exert an opposite role. It is interesting that circ-ERBIN and Erbin possess the similar expression tendency in CRC, and circRNA could increase the transcription of its parent coding genes [37]. However, the regulatory mechanism between circ-ERBIN and its linear Erbin is needed for further investigation.

Importantly, our data demonstrated the key role of circ-ERBIN in tumor angiogenesis and activation of HIF-1α signaling pathway in CRC progression. Our study uncovered the regulatory effect of circ-ERBIN on HIF-1α is not associated with alterations in HIF-1α protein stability, but correlated with increased translation of HIF-1α. Mechanistically, circ-Erbin accelerated a cap-independent protein translation of HIF-1α in CRC cells as the sponges of miR-125a-5p and miR-138-5p, which synergistically targeted 4EBP-1. It has been found that 4EBP-1 is maintained in the hyperphosphorylated state (inactive form) by the mTOR signaling to facilitate the cap-dependent translation, and is turned into hypophosphorylated state (activated) during stress such as hypoxia and nutrient starvation when mTOR activity is depressed to mediate cap-independent translation (translation of IRES-containing mRNAs) [38, 39]. Importantly, HIF-1α mRNA can utilize a dual mechanism of cap-dependent or cap-independent translation initiation [40]. Interestingly, we found that HIF-1α protein levels were significantly elevated in circ-ERBIN overexpression cells even under normoxic environment. Additionally, different activated types of phosphorylated 4EBP-1, including p-4EBP-1-Thr37/46, p-4EBP-1-Ser65, p-4EBP-1-Thr70 [41, 42], were all decreased in circ-ERBIN op stable cells. Therefore, our study indicates that circ-ERBIN might help to maintain the active status of 4EBP-1 and lead to a switch from cap-dependent to cap-independent translation in promoting HIF-1α translation in CRC progression.

Lately, circRNAs have been identified as cancer biomarkers and therapeutic targets, and most widely reported function of circRNAs is as miRNAs sponge through the circRNA-miRNA-mRNA axis. Here, we found that circ-ERBIN interacted with both miR-125a-5p and miR-138-5p in CRC cells, and circ-ERBIN had multiple binding sites for the two miRNAs. Moreover, our in vitro and in vivo results showed that circ-ERBIN-mediated promotion of proliferation and invasion could be abolished by overexpression of miR-125a-5p or miR-138-5p. Strikingly, combination of the two miRNAs had the best reversal effect in circ-ERBIN overexpressed CRC cells. Consistently, the following western blots, qRT-PCR and IHC assays all revealed the existence of circ-ERBIN/miR-125a-5p/miR-138-5p in CRC cells.

## Conclusion

In summary, Circ-ERBIN was up-regulated in clinical samples from CRC patients cancer and was positively associated with more aggressive characterization. Functionally and mechanistically, circ-ERBIN promoted the progression of CRC through circ-ERBIN/miR-125a-5p/miR-138-5p/4EBP-1 axis activated cap-independent HIF-1α translation, suggesting circ-ERBIN as a potentially promising therapeutic target for CRC.

## Supplementary Information

**Additional file 1.**

**Additional file 2.**

## Data Availability

All data generated or analyzed during this study are included in this published article. Supplementary Figures S1-S7, supplementary figure legends and Table S1 are attached.

## Abbreviations

CRC: Colorectal cancer
circRNA: Circular RNA
miRNA: microRNA
siRNAs: Short interfering RNAs
CCK-8: Cell Counting Kit-8
FISH: Fluorescence in situ hybridization
qRT-PCR: Quantitative real-time PCR
RIP: RNA immunoprecipitation
H&E: Hematoxylin and eosin
IHC: Immunohistochemical staining
