# Supplementary material for: The circular RNA circ-ERBIN promotes growth and metastasis of colorectal cancer by miR-125a-5p and miR-138-5p/4EBP-1 mediated cap-independent HIF-1α translation
Source: Mol Cancer. 2020 Nov 23;19:164. doi: 10.1186/s12943-020-01272-9 (PMC7682012; doi:10.1186/s12943-020-01272-9)
Supplement: Supplementary file 1 — Additional file 1. [file 12943_2020_1272_MOESM1_ESM.zip › Additional file1 FigureS1-S7.pptx]

## Slide 1
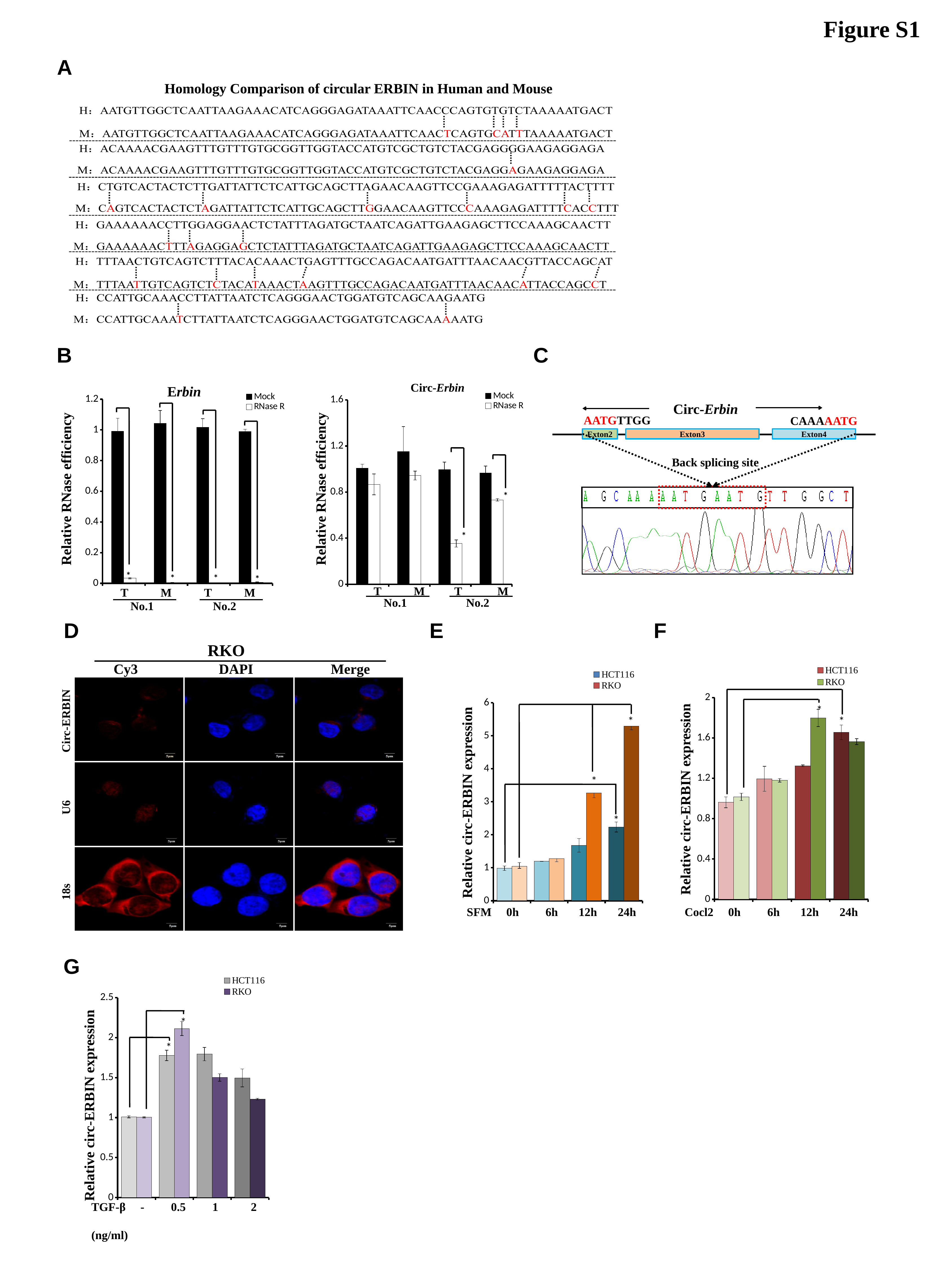

Figure S1
A
Homology Comparison of circular ERBIN in Human and Mouse
B C
Circ-Erbin
Erbin
### Chart
| Category | | |
|---|---|---|
### Chart
| Category | | |
|---|---|---|
*
*
*
*
*
*
Relative RNase efficiency
Relative RNase efficiency
T M T M
T M T M
No.1 No.2
No.1 No.2
Circ-Erbin
AATGTTGG
CAAAAATG
Exton2
Exton3
Exton4
Back splicing site
D E F
RKO
Cy3 DAPI Merge
Circ-ERBIN
U6
18s
### Chart
| Category | | |
|---|---|---|
### Chart
| Category | | |
|---|---|---|
*
*
*
*
*
Relative circ-ERBIN expression
Relative circ-ERBIN expression
SFM 0h 6h 12h 24h
Cocl2 0h 6h 12h 24h
G
### Chart
| Category | | |
|---|---|---|
*
*
Relative circ-ERBIN expression
TGF-β - 0.5 1 2
(ng/ml)

## Slide 2
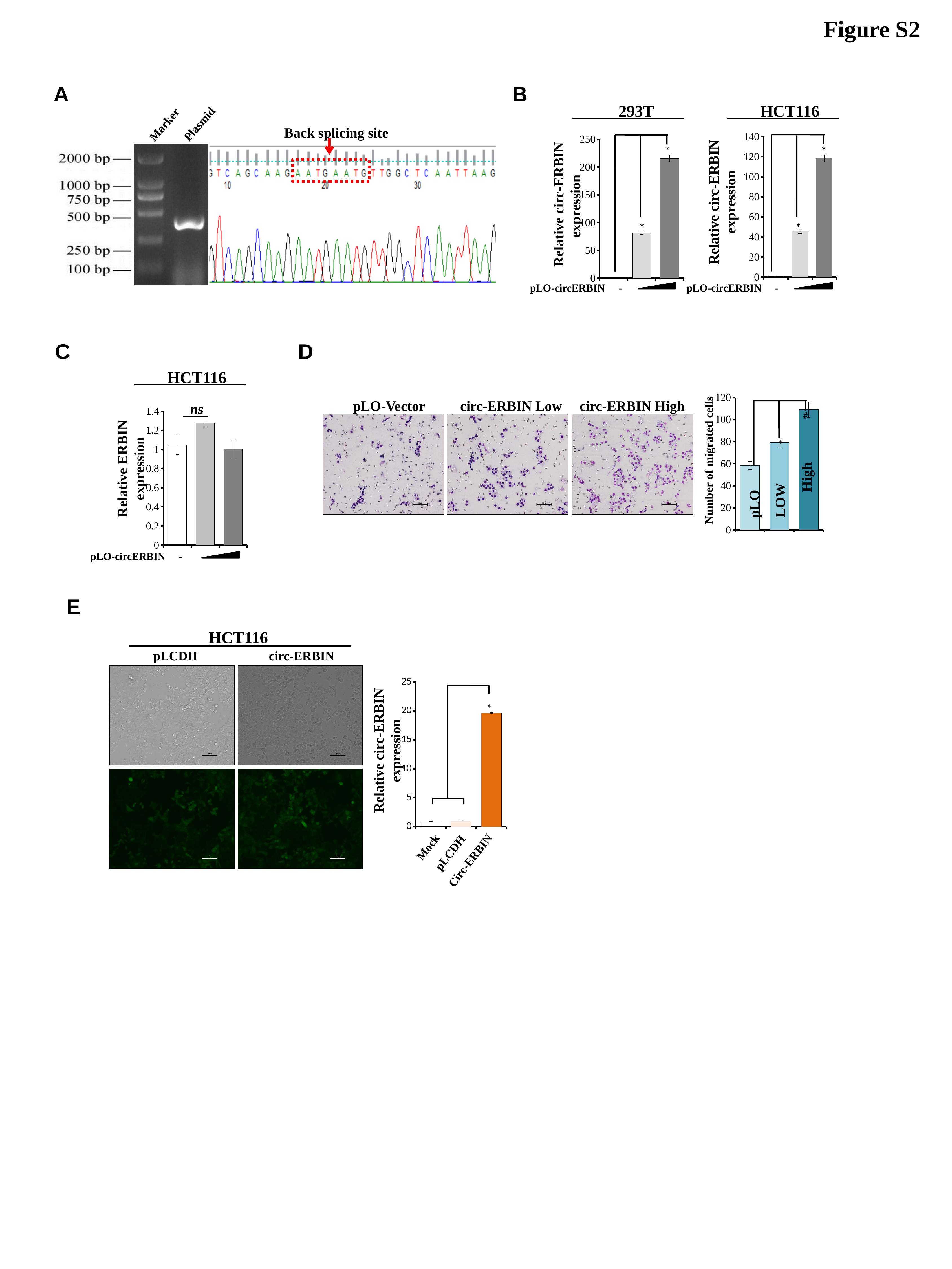

Figure S2
A B
293T
HCT116
### Chart
| Category | |
|---|---|Relative circ-ERBIN expression
### Chart
| Category | |
|---|---|Relative circ-ERBIN expression
pLO-circERBIN -
pLO-circERBIN -
Marker
Plasmid
Back splicing site
*
*
*
*
C D
HCT116
### Chart
| Category | |
|---|---|
#
*
Number of migrated cells
High
LOW
pLO
pLO-Vector circ-ERBIN Low circ-ERBIN High
ns
### Chart
| Category | |
|---|---|Relative ERBIN expression
pLO-circERBIN -
E
HCT116
pLCDH circ-ERBIN
### Chart
| Category | |
|---|---|
*
Relative circ-ERBIN expression
Mock
pLCDH
Circ-ERBIN

## Slide 3
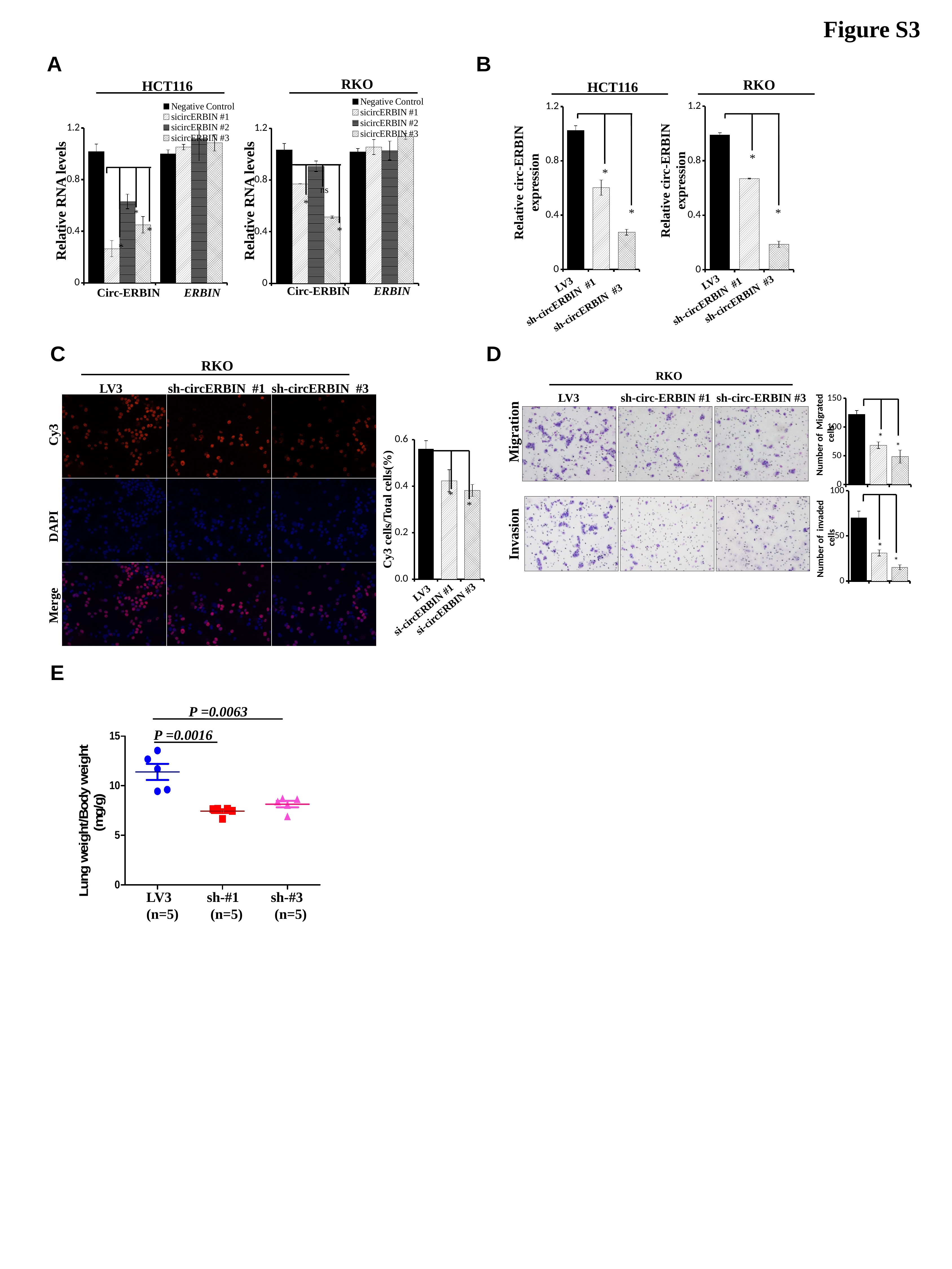

Figure S3
A B
RKO
RKO
HCT116
### Chart
| Category | |
|---|---|
### Chart
| Category | |
|---|---|
| LV3 | 0.9885229912005824 |
| sh1492#1 | 0.6683088648798425 |
| sh1492#2 | 0.18660567849078497 |
*
*
*
*
Relative circ-ERBIN expression
Relative circ-ERBIN expression
LV3
LV3
sh-circERBIN #3
sh-circERBIN #1
sh-circERBIN #1
sh-circERBIN #3
HCT116
### Chart
| Category | | | | |
|---|---|---|---|---|
### Chart
| Category | | | | |
|---|---|---|---|---|
*
*
ns
*
*
*
Relative RNA levels
Relative RNA levels
Circ-ERBIN ERBIN
Circ-ERBIN ERBIN
C D
RKO
 LV3 sh-circERBIN #1 sh-circERBIN #3
### Chart
| Category | |
|---|---|
*
*
Cy3 cells/Total cells(%)
Merge DAPI Cy3
LV3
si-circERBIN #3
si-circERBIN #1
RKO
LV3 sh-circ-ERBIN #1 sh-circ-ERBIN #3
### Chart
| Category | |
|---|---|
*
*
Number of Migrated cells
 Invasion Migration
### Chart
| Category | |
|---|---|
*
*
Number of invaded cells
E
P =0.0063
P =0.0016
LV3 sh-#1 sh-#3
(n=5) (n=5) (n=5)

## Slide 4
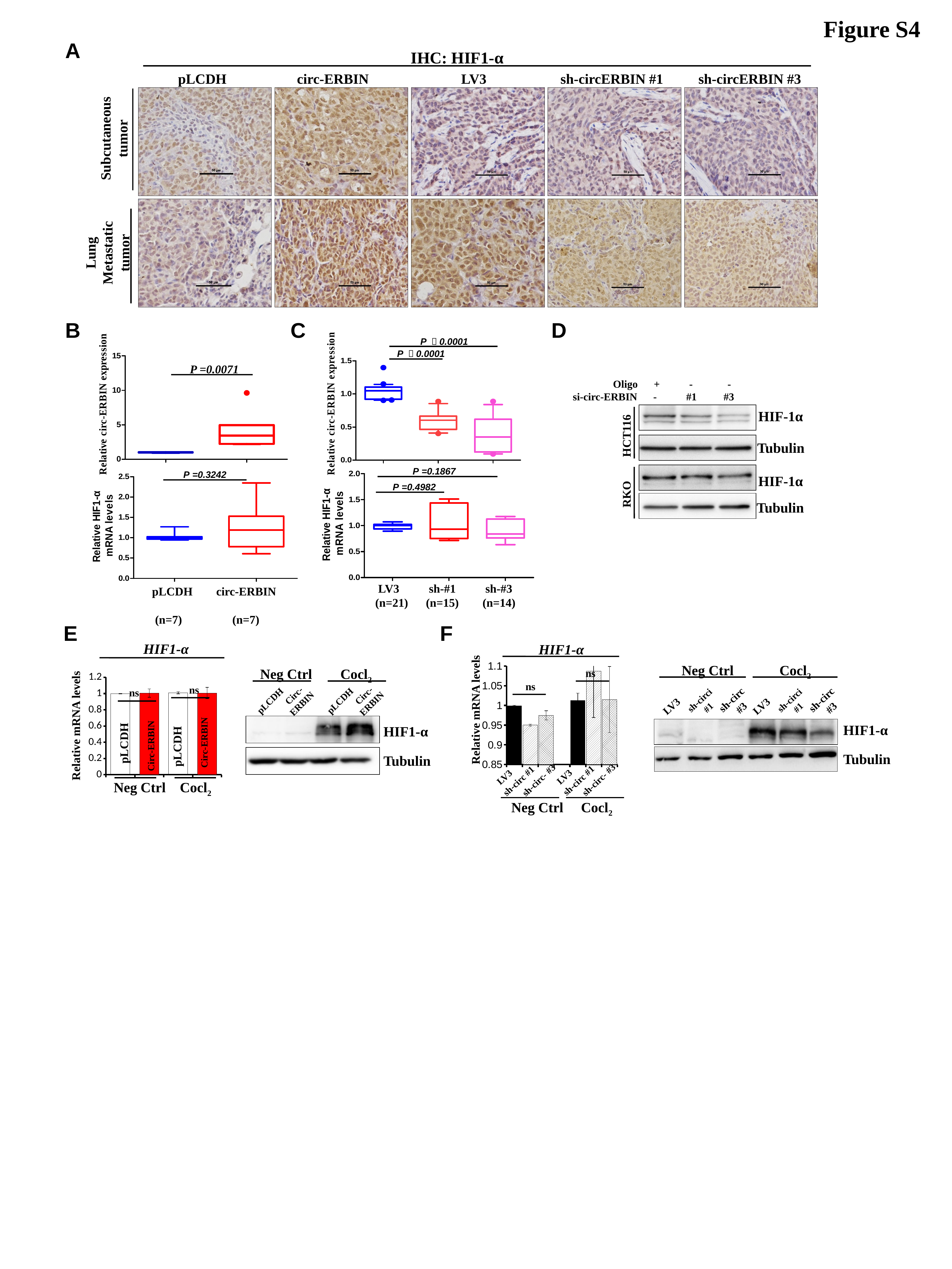

Figure S4
A
IHC: HIF1-α
pLCDH circ-ERBIN
LV3 sh-circERBIN #1 sh-circERBIN #3
Subcutaneous tumor
Lung Metastatic tumor
B C D
P ＜0.0001
P ＜0.0001
P =0.0071
 Oligo + - -
 si-circ-ERBIN - #1 #3
HIF-1α
HCT116
Tubulin
HIF-1α
RKO
Tubulin
P =0.1867
P =0.3242
P =0.4982
 LV3 sh-#1 sh-#3
(n=21) (n=15) (n=14)
 pLCDH circ-ERBIN
 (n=7) (n=7)
E F
HIF1-α
HIF1-α
### Chart
| Category | |
|---|---|Neg Ctrl Cocl2
Neg Ctrl Cocl2
Circ-
ERBIN
Circ-
ERBIN
pLCDH
pLCDH
HIF1-α
Tubulin
ns
### Chart
| Category | |
|---|---|ns
ns
ns
sh-circ
 #3
sh-circ
 #3
sh-circi
#1
sh-circi
#1
LV3
LV3
Relative mRNA levels
Relative mRNA levels
HIF1-α
Circ-ERBIN
pLCDH
Circ-ERBIN
pLCDH
Tubulin
LV3
LV3
sh-circ #1
sh-circ #1
sh-circ- #3
sh-circ- #3
Neg Ctrl Cocl2
Neg Ctrl Cocl2

## Slide 5
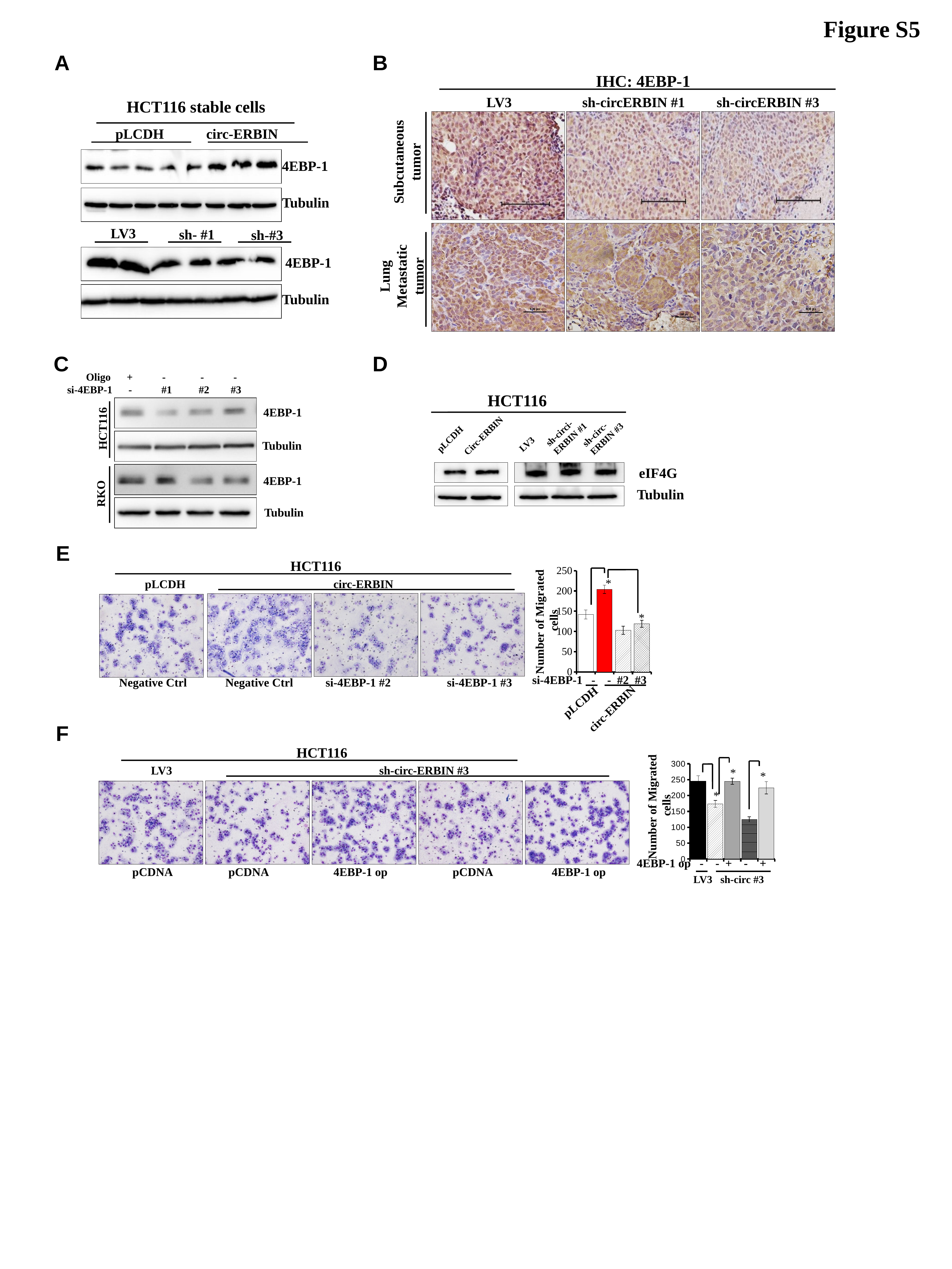

Figure S5
A B
IHC: 4EBP-1
LV3 sh-circERBIN #1 sh-circERBIN #3
Subcutaneous tumor
Lung Metastatic tumor
HCT116 stable cells
pLCDH circ-ERBIN
4EBP-1
Tubulin
LV3
sh- #1
sh-#3
4EBP-1
Tubulin
C D
 Oligo + - - -
 si-4EBP-1 - #1 #2 #3
4EBP-1
HCT116
Tubulin
4EBP-1
RKO
Tubulin
HCT116
sh-circi-
ERBIN #1
sh-circ-
ERBIN #3
Circ-ERBIN
pLCDH
LV3
eIF4G
Tubulin
E
### Chart
| Category | |
|---|---|
*
*
Number of Migrated
cells
si-4EBP-1 - - #2 #3
pLCDH
circ-ERBIN
HCT116
pLCDH circ-ERBIN
Negative Ctrl Negative Ctrl si-4EBP-1 #2 si-4EBP-1 #3
F
HCT116
### Chart
| Category | |
|---|---|
*
LV3 sh-circ-ERBIN #3
*
*
Number of Migrated
cells
4EBP-1 op - - + - +
 pCDNA pCDNA 4EBP-1 op pCDNA 4EBP-1 op
LV3 sh-circ #3

## Slide 6
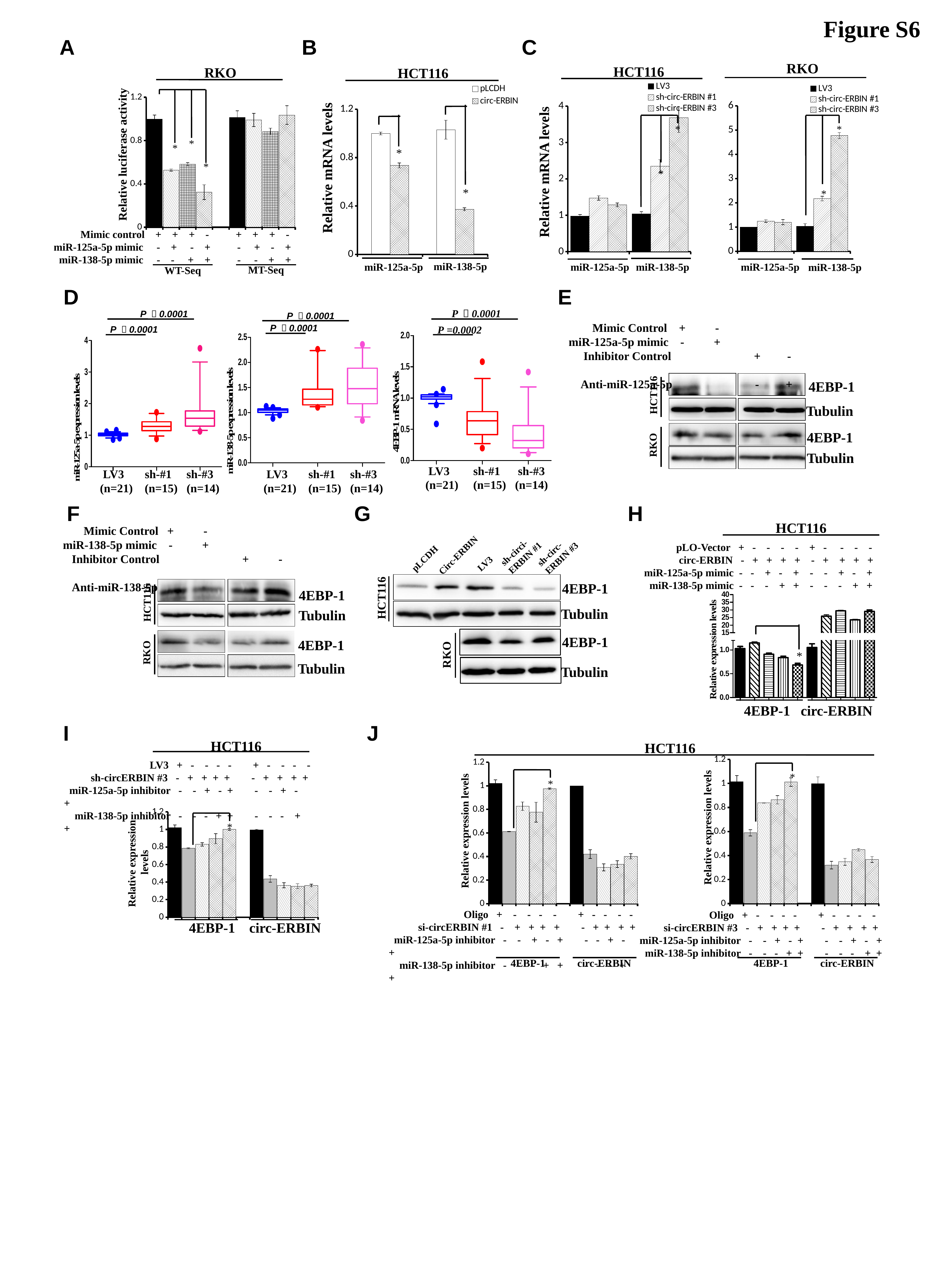

Figure S6
A B C
RKO
HCT116
RKO
*
*
*
### Chart
| Category | |
|---|---|Relative luciferase activity
 Mimic control + + + - + + + -
miR-125a-5p mimic - + - + - + - +
 miR-138-5p mimic - - + + - - + +
MT-Seq
WT-Seq
HCT116
### Chart
| Category | | | |
|---|---|---|---|
### Chart
| Category | | |
|---|---|---|
### Chart
| Category | | | |
|---|---|---|---|
*
*
*
*
*
*
Relative mRNA levels
Relative mRNA levels
miR-138-5p
miR-125a-5p
miR-138-5p
miR-125a-5p
miR-125a-5p
miR-138-5p
D E
P ＜0.0001
P ＜0.0001
P ＜0.0001
P ＜0.0001
P =0.0002
P ＜0.0001
 LV3 sh-#1 sh-#3
(n=21) (n=15) (n=14)
 LV3 sh-#1 sh-#3
(n=21) (n=15) (n=14)
 LV3 sh-#1 sh-#3
(n=21) (n=15) (n=14)
 Mimic Control + -
miR-125a-5p mimic - +
 Inhibitor Control + -
 Anti-miR-125a-5p - +
4EBP-1
HCT116
Tubulin
4EBP-1
RKO
Tubulin
F G H
HCT116
 pLO-Vector + - - - - + - - - -
 circ-ERBIN - + + + + - + + + +
 miR-125a-5p mimic - - + - + - - + - +
 miR-138-5p mimic - - - + + - - - + +
 4EBP-1 circ-ERBIN
*
 Mimic Control + -
 miR-138-5p mimic - +
 Inhibitor Control + -
 Anti-miR-138-5p - +
4EBP-1
HCT116
Tubulin
4EBP-1
RKO
Tubulin
sh-circi-
ERBIN #1
sh-circ-
ERBIN #3
Circ-ERBIN
pLCDH
LV3
4EBP-1
HCT116
Tubulin
4EBP-1
RKO
Tubulin
I J
HCT116
 LV3 + - - - - + - - - -
 sh-circERBIN #3 - + + + + - + + + +
 miR-125a-5p inhibitor - - + - + - - + - +
 miR-138-5p inhibitor - - - + + - - - + +
### Chart
| Category | |
|---|---|Relative expression
 levels
 4EBP-1 circ-ERBIN
*
HCT116
### Chart
| Category | |
|---|---|
### Chart
| Category | |
|---|---|
Relative expression levels
Relative expression levels
 Oligo + - - - - + - - - -
 si-circERBIN #1 - + + + + - + + + +
 miR-125a-5p inhibitor - - + - + - - + - +
 miR-138-5p inhibitor - - - + + - - - + +
 Oligo + - - - - + - - - -
 si-circERBIN #3 - + + + + - + + + +
 miR-125a-5p inhibitor - - + - + - - + - +
 miR-138-5p inhibitor - - - + + - - - + +
 4EBP-1 circ-ERBIN
 4EBP-1 circ-ERBIN
*
*

## Slide 7
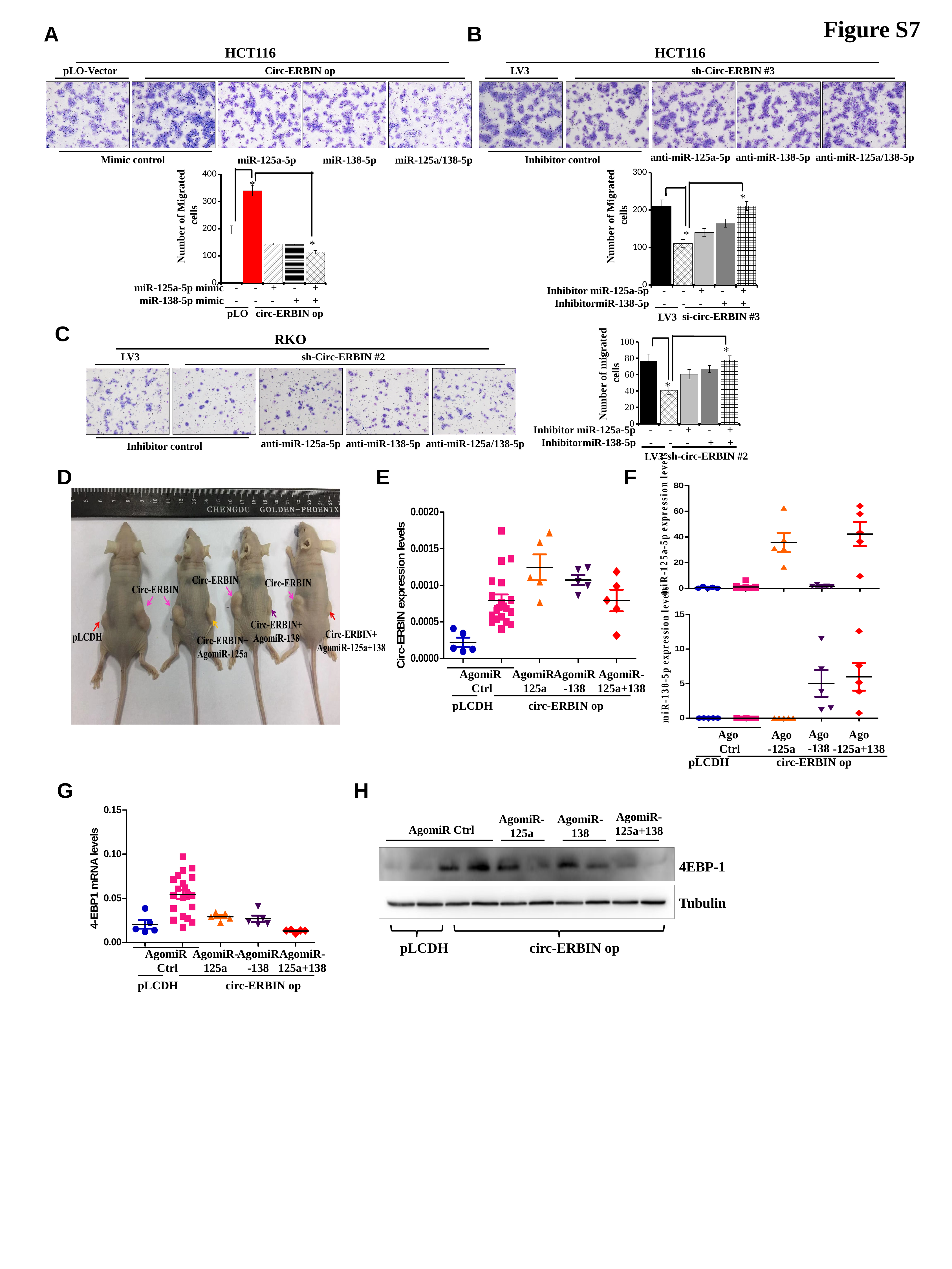

Figure S7
A B
HCT116
pLO-Vector
Circ-ERBIN op
Mimic control
miR-125a-5p miR-138-5p miR-125a/138-5p
### Chart
| Category | |
|---|---|
*
*
Number of Migrated
 cells
miR-125a-5p mimic - - + - +
 miR-138-5p mimic - - - + +
pLO
circ-ERBIN op
HCT116
LV3
sh-Circ-ERBIN #3
anti-miR-125a-5p anti-miR-138-5p anti-miR-125a/138-5p
Inhibitor control
### Chart
| Category | |
|---|---|
*
*
Number of Migrated
 cells
Inhibitor miR-125a-5p - - + - +
 InhibitormiR-138-5p - - - + +
si-circ-ERBIN #3
LV3
C
RKO
LV3
sh-Circ-ERBIN #2
anti-miR-125a-5p anti-miR-138-5p anti-miR-125a/138-5p
Inhibitor control
### Chart
| Category | |
|---|---|
*
*
Number of migrated
 cells
Inhibitor miR-125a-5p - - + - +
 InhibitormiR-138-5p - - - + +
sh-circ-ERBIN #2
LV3
D E F
AgomiR
Ctrl
AgomiR-125a+138
AgomiR-125a
AgomiR
-138
pLCDH circ-ERBIN op
Ago
-138
Ago
Ctrl
Ago
-125a+138
Ago
-125a
pLCDH circ-ERBIN op
G H
AgomiR
Ctrl
AgomiR-125a+138
AgomiR-125a
AgomiR
-138
pLCDH circ-ERBIN op
AgomiR-125a+138
AgomiR-125a
AgomiR-138
AgomiR Ctrl
4EBP-1
Tubulin
pLCDH circ-ERBIN op
