# Supplementary material for: The circular RNA circ-ERBIN promotes growth and metastasis of colorectal cancer by miR-125a-5p and miR-138-5p/4EBP-1 mediated cap-independent HIF-1α translation
Source: Mol Cancer. 2020 Nov 23;19:164. doi: 10.1186/s12943-020-01272-9 (PMC7682012; doi:10.1186/s12943-020-01272-9)
Supplement: Supplementary file 1 — Additional file 1. [file 12943_2020_1272_MOESM1_ESM.zip › Supplementary figure legends.docx]

**< Supplementary figure legends>**

**Additional file2: Figure S1: The identification of the circ-ERBIN.**

***(A).*** Sequence alignment for circ-ERBIN between human and mouse. ***(B).*** qRT–PCR for the expression of *ERBIN* (left) and circ-ERBIN (right) RNA in the tumor samples of a mouse model of CRC metastasis treated with RNase R. All the qRT-PCR experiments were normalized using GAPDH as an endogenous control. Data are expressed as the mean±SD, *, P＜0.05. ***(C).*** Sanger sequencing following qPCR conducted using the divergent primers confirmed the “head-to-tail” splicing of circ-ERBIN in the mice tissues. ***(D).*** RNA fluorescence in situ hybridization for circ-ERBIN in RKO cells. Nuclei were stained with DAPI. ***(E-G).***HCT116 and RKO cells were treated with SFM***(E)***, Cocl2***(F)*** or TGF-β***(G)*** as represented in the figure and followed by qRT-PCR experiments using circ-ERBIN divergent primers. Data are expressed as the mean±SD, *, P＜0.05.

**Additional file2: Figure S2: Overexpression of circ-ERBIN promotes proliferation, migration, invasion of CRC in vitro and in vivo.**

***(A).*** Sanger sequencing following qRT-PCR conducted using the indicated divergent primers confirmed the synthetic circRNA expression plasmid. ***(B-C).*** Relative RNA levels of *ERBIN* and circ-ERBIN were analyzed by qRT-PCR and normalized to the value detected in the mock group, respectively. All the RT-PCR experiments were normalized using GAPDH as an endogenous control. ***(D).*** Cells were transfected with circ-ERBIN plasmids, and were analyzed for their in vitro migrativeness using transwell chambers. Cells were stained by Giemsa’s staining and visualized under a phase-contrast microscope. ***(E).*** Photos for HCT116 stable cells to show the infection efficiency. Data are means ± SD. *, *P*＜0.05 vs pLCDH.

**Additional file2: Figure S3: Circ-ERBIN knockdown suppresses CRC cells proliferation, migration and invasion in vitro and in vivo.**

***(A).*** qRT-PCR performed in si-circ-ERBIN transfected HCT116 and RKO cells, respectively. ***(B).*** HCT116 and RKO cells transfected with empty vector (LV3) or vectors containing sh-circ-ERBIN sequence. Total RNAs were extracted from circ-ERBIN knockdown cell lines. All the qRT-PCR experiments were normalized using GAPDH as an endogenous control.  ***(C).*** Edu assays showed decreased proliferation in sh-circ-ERBIN stable cells.  ***(D).*** Transwell migration and invasion experiments in RKO sh-circ-ERBIN stable cells. ***(E).*** Lung weight/Body weight value (mg/g) was calculated in the metastasis model of CRC. Data are means ± SD. *, *P*＜0.05 vs LV3.

**Additional file2: Figure S4: Circ-ERBIN exerts an oncogenic role via alleviating HIF-1α levels in CRC cells.**

1. IHC experiments in subcutaneous tumors and lung metastases formed by circ-ERBIN op or knockdown cells. IHC revealed that HIF-1α staining significantly enhanced or suppressed in overexpressed group or knockdown group, compare to the control group, respectively. ***(B-C).*** qRT-PCR experiments were performed using subcutaneous tumor tissues formed by circ-ERBIN op ***(B)*** or sh-circ-ERBIN ***(C)*** cells. ***(D).*** HCT116 and RKO cells were transfected with siRNAs targeting circ-ERBIN. Western blot experiments were performed. ***(E-F).*** Cells stably overexpressed or knockdown of circ-ERBIN were treated with Cocl_2_ or not, qRT-PCR and western blot experiments were performed.

**Additional file2: Figure S5: Circ-ERBIN elevates the expression of HIF-1α via 4EBP-1.**

***(A).*** Western blot experiments were performed using subcutaneous tumor tissues formed by stably overexpressed or knockdown of circ-ERBIN cell lines, respective antibodies were used. ***(B).***Circ-ERBIN knockdown (LV3/sh-circERBIN #1/sh-circERBIN #3) stable cells were used to perform subcutaneous tumor model and tail vein injection metastasis model, respectively. Immunohistochemical staining of 4EBP-1 were presented. Scale bars, 100μm. ***(C).*** HCT116 and RKO cells were transfected with 4EBP-1 siRNAs, and western blots experiments were used to determine the efficiency of the siRNAs. ***(D).*** Western blot experiments were performed using stably overexpressed or knockdown of circ-ERBIN cell lines, eIF4G antibody was used. ***(E).*** HCT116 circ-ERBIN stably overexpressed cells alone or transfected with 4EBP-1 siRNAs for 2 days followed by the analysis of cell migration using transwell assay. ***(F).*** HCT116 sh-circ-ERBIN cells alone or transfected with 4EBP-1 plasmids for 2 days followed by the analysis of cell migration using Transwells. For transwell assays, cells were stained by Giemsa’s staining and visualized under a phase-contrast microscope. Data are presented as means ± SD. Student’s t-test was used. *, *P*＜0.05.

**Additional file2: Figure S6: Circ-ERBIN elevates 4EBP-1 expression by sponging miR-125a-5p and miR-138-5p.**

***(A).*** Luciferase activity of LUC-circ-ERBIN WT or LUC-circ-ERBIN Mutant in RKO cells after co-transfection with miR-125a-5p or/and miR-138-5p mimics. ***(B-C).*** qRT-PCR performed in circ-ERBIN overexpression ***(B)*** and knockdown ***(C)*** stable cells, respectively. ***(D).*** HCT116 cells stably knockdown of circ-ERBIN (sh-circ-ERBIN #1 and sh-circ-ERBIN #3) or empty vector (LV3) were injected subcutaneously into nude mice for 4 weeks. Tumors were taken out and qRT-PCR was performed. Data are means ± SD. P＜0.05. ***(E-F).*** miR-125a-5p and miR-138-5p mimics or inhibitor were transfected into HCT116 or RKO cells, respectively. Protein levels of 4EBP-1 were analyzed. ***(G).*** Western blot experiments were performed using HCT116 and RKO circ-ERBIN stably overexpressed or knockdown cell lines, respective antibodies were used. ***(H).*** HCT116 cells were transfected with circ-ERBIN plasmids alone or together with either miR-125a-5p or miR-138-5p mimics, or altogether for 2 days, and followed by qRT-PCR experiments. ***(I).*** miR-125a-5p or miR-138-5p inhibitor was transfected alone or together into HCT116 sh-circ-ERBIN stable cell lines. qRT-PCR experiments were performed to analyze the RNA levels of 4EBP-1 and circ-ERBIN. ***(J).*** HCT116 cells were transfected with si-circ-ERBIN siRNAs alone or together with miR-125a-5p or miR-138-5p inhibitor for 2 days, and followed by qRT-PCR experiments. Data are means ± SD. *, P＜0.05

**Additional file2: Figure S7: Circ-ERBIN accelerates the growth and metastasis of CRC by activating HIF-1α signaling through the circ-ERBIN/miR-125a-5p/miR-138-5p/4EBP-1 pathway.**

***(A).*** HCT116 cells were transfected with circ-ERBIN plasmids alone or together with miR-125a-5p or miR-138-5p mimics for 2 days followed by the analysis of cell migration using Transwells. ***(B-C).*** MiR-125a-5p or miR-138-5p inhibitor were transfected alone or together into HCT116 ***(B)*** or RKO ***(C)*** sh-circ-ERBIN #3 stable cell lines for 2 days followed by the analysis of cell migration using transwell assays.

Cells were stained by Giemsa’s staining and visualized under a phase-contrast microscope. For transwell assays, data are presented as means ± SD. Student’s t-test was used. ***(D-G).*** HCT116 stably expressed circ-ERBIN or pLCDH vector were injected into BALB/C nude mice (n=5 for each group). AgomiRs were injected into tumors as represented and pictures were presented ***(D).*** Tumors were taken out and qRT-PCR experiments were performed to test the RNA levels of circ-ERBIN ***(E)***, miR-125a-5p ***(F)***, miR-138-5p ***(F)*** and 4EBP-1 ***(G)***. ***(H).*** 4EBP-1 protein levels were analyzed using the subcutaneous tumors described in figure S7D.
